# AKT regulates NPM dependent ARF localization and p53^mut^ stability in tumors

**DOI:** 10.18632/oncotarget.2178

**Published:** 2014-07-08

**Authors:** Garth Hamilton, Aswin G. Abraham, Jennifer Morton, Oliver Sampson, Dafni E. Pefani, Svetlana Khoronenkova, Anna Grawenda, Angelos Papaspyropoulos, Nigel Jamieson, Colin McKay, Owen Sansom, Grigory L. Dianov, Eric O'Neill

**Affiliations:** ^1^ Cancer Research UK/MRC Oxford Institute, Department of Oncology, University of Oxford, Old Road Campus, Roosevelt Drive, UK; ^2^ Beatson Institute for Cancer Research, Garscube Estate, Switchback Road, Glasgow, UK; ^3^ West of Scotland Pancreatic Unit and University Department of Surgery, Glasgow Royal Infirmary, Alexandra Parade. Glasgow

**Keywords:** ARF, p53, AKT, Pancreatic, Cance

## Abstract

Nucleophosmin (NPM) is known to regulate ARF subcellular localization and MDM2 activity in response to oncogenic stress, though the precise mechanism has remained elusive. Here we describe how NPM and ARF associate in the nucleoplasm to form a MDM2 inhibitory complex. We find that oligomerization of NPM drives nucleolar accumulation of ARF. Moreover, the formation of NPM and ARF oligomers antagonizes MDM2 association with the inhibitory complex, leading to activation of MDM2 E3-ligase activity and targeting of p53. We find that AKT phosphorylation of NPM-Ser48 prevents oligomerization that results in nucleoplasmic localization of ARF, constitutive MDM2 inhibition and stabilization of p53. We also show that ARF promotes p53 mutant stability in tumors and suppresses p73 mediated p21 expression and senescence. We demonstrate that AKT and PI3K inhibitors may be effective in treatment of therapeutically resistant tumors with elevated AKT and carrying gain of function mutations in p53. Our results show that the clinical candidate AKT inhibitor MK-2206 promotes ARF nucleolar localization, reduced p53^mut^ stability and increased sensitivity to ionizing radiation in a xenograft model of pancreatic cancer. Analysis of human tumors indicates that phospho-S48-NPM may be a useful biomarker for monitoring AKT activity and *in vivo* efficacy of AKT inhibitor treatment. Critically, we propose that combination therapy involving PI3K-AKT inhibitors would benefit from a patient stratification rationale based on ARF and p53^mut^ status.

## INTRODUCTION

The phosphatidylinositol 3 kinase (PI3K)-AKT signaling cascade is a vital mediator of essential cellular activities including proliferation and survival [1]. Mutations, amplification and deletions of the upstream regulators of AKT are among the most frequent somatic events in cancer [2, 3]. Consequently, deregulation of AKT is a major factor enhancing both oncogenesis and resistance to treatment in many human malignancies [4, 5]. Alteration of upstream pathway components, such as activating mutations in RAS, PI3K or loss of PTEN, can be the primary oncogenic event leading to therapeutic failure[2, 6]. However, it is the activation of AKT that is proposed to modulate cell death responses to therapeutic agents and mediate resistance [3, 7]. Not surprisingly, the aim of regaining sensitivity to various therapies has focused attention on targeting the PI3K-AKT pathway [8]. PI3K and AKT inhibitors, such as PI-103 and MK-2206 have been investigated as single agents, but their potential as combination agents in specific patient cohorts is anticipated to be where the greatest effectiveness of these agents will be identified [9, 10].

TP53 is an important mediator of cell death responses to commonly used therapeutic agents that elicit DNA damage [11-13]. The potent tumor suppressor functions of p53 require that the activity of this protein is under tight control to prevent unnecessary induction of apoptosis or cellular senescence [14-16]. In untransformed cells p53 is targeted for proteasomal degradation by the E3 ubiquitin ligase MDM2 [17]. The activity of MDM2 is antagonized by p14^ARF^ (p19^ARF^ in mouse – hereafter ARF), a product of the *INK4A/ARF* locus [18]. In untransformed cells, ARF mediated inhibition of MDM2 and subsequent p53 activation is important in the induction of p53 tumor suppressor activities, including the activation of cellular senescence following oncogenic insult [19-22].

The functional inactivation of the p53 pathway, either through mutation of p53 itself or the deregulation of upstream regulatory elements is a universal feature of human cancer [16, 23]. Indeed somatic mutations of p53 are found in nearly half of all human cancers [24, 25]. Recently, mutant p53 (p53^mut^) has been demonstrated to respond to many of the same stimuli that promote wild type p53 stabilization, indicating that wild type and mutant p53 share similar regulatory mechanisms [26].

A hallmark of tumors with missense mutations in p53 is the accumulation of p53^mut^ within tumor tissue, which contributes to the many gain of function phenotypes attributed to p53^mut^ [24, 25]. Furthermore, many genetic modifications found in cancer including RAS mutation, c-MYC activation, p16^INK4A^ loss or PML deletion have been demonstrated to stabilize p53^mut^ [26-28]. In normal tissue, mutation of p53 alone is in itself not sufficient to promote p53^mut^ accumulation. Furthermore, as tumors originating from p53^mut^ mice do not accumulate p53^mut^ to the same degree, it suggests that there may be some degree of tissue specificity regarding the mechanisms which contribute to p53^mut^ stability [27, 29, 30]. Growing evidence indicates that tumor cells must also acquire additional mutations for p53^mut^ to overcome regulatory mechanisms that normally protect against inappropriate p53 accumulation in normal cells [24, 25, 27, 31, 32]. Although MDM2 has been demonstrated to restrict the stabilization of p53^mut^ [27] the molecular determinants and pathways that promote p53^mut^ stabilization remain to be fully determined and have the potential to offer new therapeutic avenues to the treatment of tumors harboring p53^mut^.

In human tumors ARF is infrequently mutated and predominately inactivated through promoter methylation or transcriptional inactivation. While ARF activity maybe lost due to mutations at the INK4A/ARF locus, a number of studies have shown that they can be mutually exclusive, where INK4A maybe lost while sparing ARF [33]. Although ARF expression has been reported in cell lines with p53^mut^ and has been suggested to protect p53^mut^ from degradation [34, 35], it is unclear whether ARF contributes to the regulation of p53^mut^.

ARF itself is regulated by nucleophosmin (NPM), a predominantly nucleolar protein, which due to the presence of multiple sub-cellular localization signals shuttles between the nucleolus, nucleoplasm and cytoplasm [36, 37]. NPM is required for both ARF stability and targeting to the nucleolus [38-41]. Indeed NPM has been proposed to sequester ARF in the nucleolus, preventing it from inhibiting MDM2 [41, 42]. The importance of NPM in regulating ARF stability is highlighted by the frequent somatic mutation in acute myeloid leukemia (NPMc) which increases the trafficking of NPM to the cytoplasm resulting in increased ARF turnover [43, 44]. Given that ARF has been proposed to inhibit MDM2 in the nucleoplasm and nucleolar localization of ARF protects it from degradation, a conundrum exists regarding the mechanism that allows NPM to restrict ARF nucleolar accumulation and thereby allow MDM2 inhibition [41, 45-48]

In this article we identify that AKT regulates both ARF stability and localization at the nucleolus. We find that AKT phosphorylation of NPM-Ser48 inhibits NPM oligomerisation and localization at the nucleolus. This in turn promotes ARF mediated inhibition of MDM2 in the nucleoplasm. Importantly, AKT mediated promotion of ARF localization in the nucleoplasm facilitates oncogenesis by promoting p53^mut^ stability and dominant negative suppression of the DNA damage response, thereby contributing to therapeutic resistance. We provide molecular evidence for resensitization of tumors by PI3K-AKT inhibitors. Most importantly, our findings indicate that AKT mediated resistance associates with *INK4A/ARF* status and p53^mut^, therefore identifying a screening rationale for the patient population in which PI3K-AKT inhibitors are most likely to display efficacy.

## RESULTS

### AKT phosphorylation of NPM-Ser48 regulates NPM oligomerization

**Fig.1:**
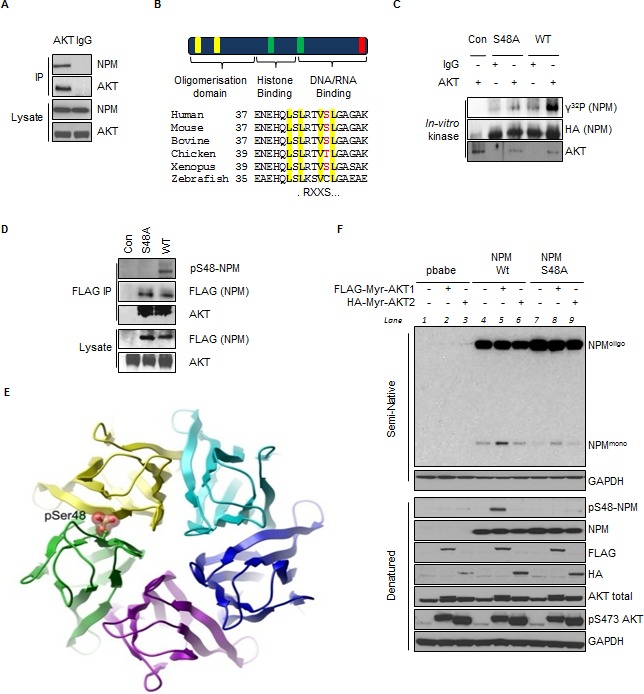
AKT Phosphorylates NPM on Serine 48 and Regulates NPM Quaternary Structure (A) AKT or IgG immunoprecipitates and whole cell lysates from T24 cells were probed with indicated antibodies. (B) (Top) Domain structure of NPM highlighting the nuclear export signals (NES) (yellow), nuclear localization signals (green) and nucleolar localization signal (red); (Bottom) Sequence alignment illustrating the conservation of Ser48 within the first NES. (C) *In-vitro* kinase assay of immunopurified AKT or IgG control with NPM mutants in the presence of radiolabeled (γ^32^P) ATP as indicated. (D) Anti-FLAG immunoprecipitates from T24 cells transfected with empty vector (con), FLAG-NPM-WT or FLAG-NPM-S48A. Immunoprecipitates and whole cell lysates were probed with the indicated antibodies. (E) Ribbon diagram of the NPM pentameric ring (top view) showing a space filling model of phosphorylation at Ser48, highlighting a steric clash with the neighbouring subunit. Model is based on PDB entry 2P1B. (F) *Npm−/−, p53−/−* double null MEF were infected with pBabe retrovirus expressing FLAG-tagged-myristoylated (myr)-AKT1 or HA-tagged-myr-AKT2 in combination with NPM-WT or NPM-S48A as indicated. NPM oligomerisation status was determined by mild semi native gel electrophoresis (Fig. S1H) and denatured lysates, which were probed with the indicated antibodies.

NPM was identified by mass spectrometry in AKT immunoprecipitates and verified to associate with active AKT by western blot (Fig. 1A, S1A and S1B). Although other groups have reported an interaction between AKT and NPM [49], a role for this association has not been addressed. In T24 cells with oncogenically active AKT, NPM is readily detectable using a pan-AKT substrate antibody but not in the presence of the PI3-kinase inhibitor PI-103, indicating that NPM was a possible AKT substrate (Fig. S1C). NPM contains a single non-consensus (RxxS) AKT substrate motif at position Ser48 within the N-terminal oligomerisation domain (Fig. 1B) and the RxxS motif has previously been reported to function as a *bona-fide* AKT substrate recognition motif in CDK2 [50]. We confirmed that AKT specifically phosphorylated NPM on Ser48 by *in-vitro* kinase assay (Fig. 1C). Phosphorylated NPM, but not a NPM-S48A derivative, can be detected with a specific phospho-peptide antibody (pS48-NPM) (Fig. 1D and S1D) and furthermore, NPM-Ser48 could be phosphorylated by AKT in response to EGF stimulation (Fig. S1E).

In order to determine how AKT mediated phosphorylation regulates NPM function, we examined the crystal structure of the NPM N-terminal oligomerisation domain [51]. The structure of the monomer indicates that phospho-Ser48 can be accommodated (Fig. S1F) but is incompatible with incorporation into the pentameric ring due to steric clashes at the monomer-monomer interface (Fig. 1E). To address if phosphorylation of NPM-S48 regulated NPM oligomerisation, *Npm^−/−^*; *p53^−/−^* MEF (hereafter *Npm*^−/−^ MEF) were reconstituted with NPM-WT, non-phosphorylatable NPM-S48A or phosphomimetic NPM-S48E derivatives (Fig. 1F and S1G) and lysates from reconstituted *Npm^−/−^* MEF were resolved under native conditions. In agreement with previous studies [52], NPM was detected as both a monomer and an oligomer by appropriate semi-native electrophoresis conditions (Fig. 1F, lane 4, Fig. S1G and S1H). The non-phosphorylatable NPM-S48A appeared more oligomeric and the phosphomimetic mutant, NPM-S48E, although less stable under stronger denaturing conditions, was exclusively monomeric (Fig. S1G), as has previously been reported for NPM mutants that cannot oligomerize [52]. Interestingly, we observed that NPM-S48A oligomers were more resistant to mild denaturing conditions (Fig. S1H). Recent mathematical modeling predicted that Ser48 should be important for controlling the NPM monomer:oligomer equilibrium [53]. As NPM oligomerization is incompatible with phospho-S48-NPM, we wished to confirm whether AKT could inhibit oligomerisation as suggested by the NPM-S48E phospho-mimetic mutant (Fig. S1G).

To determine the effect of AKT activation on NPM oligomerization we examined the reconstituted *Npm^−/−^* MEFs in the presence or absence of constitutively active AKT1 or AKT2 (Fig. 1F). In the presence of myr-AKT1, we observed an increased monomeric NPM fraction, corresponding with increased NPM-S48 phosphorylation, implying that phosphorylation prevents NPM oligomers in a Ser48 dependent manner (Fig. 1F, lanes 4-6). Co-expression of K-Ras^V12^, thereby enhancing PI3K-AKT activity, similarly disrupts pentameric NPM but not NPM-S48A (data not shown). Thus, oncogenic activation of AKT disrupts NPM quaternary dynamics via phosphorylation of Ser48.

### Phosphorylation of NPM-Ser48 controls NPM and ARF localization

**Fig.2:**
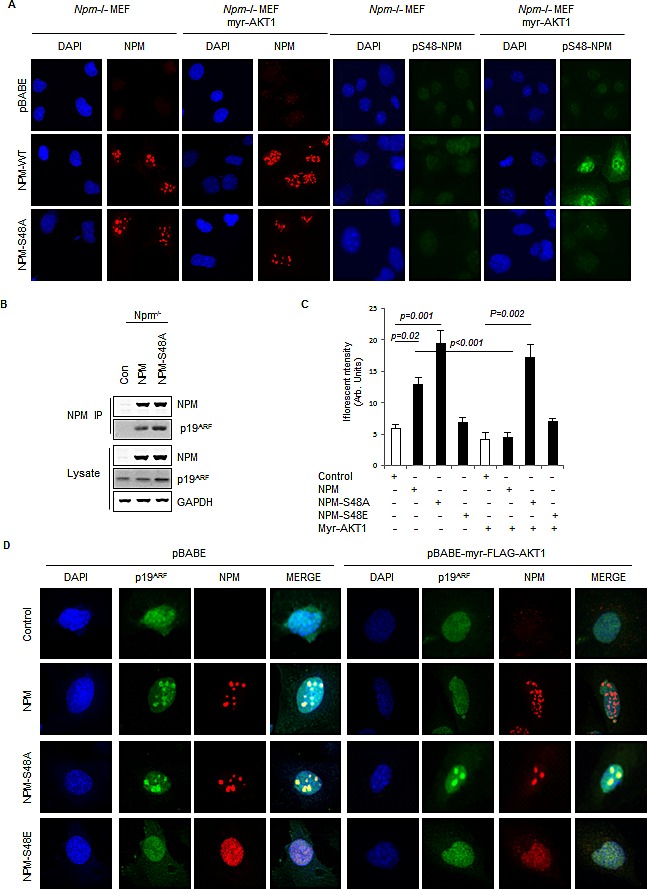
Phosphorylation of NPM-Ser48 regulates the localization of NPM and p19A (A) *Npm−/−, p53−/−*double null MEF were infected with pBabe retrovirus expressing FLAG-tagged-myr-AKT1 in combination with NPM-WT or NPM-S48A as indicated. Cells were fixed and stained with DAPI and anti-NPM (left) or anti-phospho-S48-NPM (pS48-NPM). (B) NPM immunoprecipitates and whole cell lysates from *Npm−/−;p53−/−* MEFs expressing human NPM or NPM-S48A were probed with the indicated antibodies.(C) Graph, quantification of p19ARF staining intensity in confocal images using ImageJ. (D) *Npm−/−, p53−/−*double null MEF were infected with pBABE retrovirus expressing FLAG-tagged-myr-AKT1 in combination with NPM-WT, NPM-S48A or S48E as indicated. Cells were fixed and stained with DAPI, anti-NPM and anti p19ARF.

NPM isolated from the nucleolus is predominantly oligomeric in nature [54] and as phosphorylation of Ser48 influences NPM oligomerisation we next wished to address if NPM-Ser48 was regulating NPM localization. Ser-48 lies within a characterized nuclear export sequence (NES) (Fig. 1B) [55], and interestingly mutation of either NPM NES has been shown to impair the nucleolar localization of NPM [56]. In order to address this, *Npm^−/−^* MEF were reconstituted with NPM-WT or NPM-S48A and levels of phospho-S48-NPM were determined by immunofluorescence (Fig. 2A). Phosphorylation was only detected in cells expressing wild type NPM and constitutively active myr-AKT1, but not in NPM-S48A expressing cells (Fig. 1F and 2A). In contrast to total NPM staining in cells expressing NPM or NPM-S48A, phospho-S48 NPM was not localized to the nucleoli and was instead distributed throughout the nucleoplasm and cytoplasm (Fig. 2A), agreeing with previous reports that non-oligomeric NPM is deficient in nucleolar targeting [52, 56]. This suggests that phosphorylation of NPM-Ser48 is a physiological signal that directs NPM localization via the regulation of NPM oligomerisation.

Since phosphorylation of NPM-Ser48 appears to dictate both NPM oligomerisation and localization, we wished to address whether this mechanism may also affect NPM interacting proteins that are functionally controlled by trafficking to and from the nucleolus [36]. Importantly, NPM has been widely observed to regulate both ARF protein stability and localization [38, 40-42, 52]. Moreover, site directed mutants of NPM that restrict oligomerization also perturb ARF association and nucleolar targeting [39, 52, 56], suggesting that phosphorylation of NPM-Ser48 by AKT may also impact ARF localization and stability. To test this, we transfected *Npm*^−/−^ MEFs with both Flag-NPM and Flag-NPM-S48A and determined the ability of these derivatives to co-immunoprecipitate ARF (Fig. 2B). We found that while both derivatives are capable of binding ARF, cells expressing NPM-S48A have elevated total ARF protein levels, indicative of enhanced ARF stabilization (Fig. 2B). Thus, Ser48-NPM does not directly influence the ability of NPM to associate with ARF but results in differential ARF stability. Increased ARF stability is associated with nucleolar protection from ubiquitin ligase mediated degradation [57], suggesting that nucleolar localization of ARF may be affected. We next examined ARF localization in *Npm*^−/−^ MEFs and found that endogenous ARF displays a diffuse nucleoplasmic staining, but reconstitution with NPM-WT restricts ARF localization to distinct punctuate nucleolar foci [38] and increases ARF immunofluorescence intensity (Fig. 2C and 2D). Expression of NPM-S48A results in an elevation of ARF staining (Fig. 2C), which suggests, together with Fig. 2B, that higher protein levels are associated with localization to the nucleolus (Fig. 2D). To determine whether constitutive phosphorylation on Ser48 would have the opposite effect on ARF stability and localization, we expressed the phosphomimetic derivative, NPM-S48E that displays similar localization to phospho-S48-NPM (Fig. 2A and 2C) and also fails to associate into pentamers (Fig. S1G). This derivative is less stable than NPM-WT making direct comparisons on overall ARF stability in lysates difficult. However, we clearly see that cells expressing NPM-S48E are unable to promote any nucleolar NPM or ARF (Fig. 2D). Expression of myr-AKT in these cells results in increased monomers and disruption of NPM nucleolar signal of the NPM-WT, although oligomers and foci are still visible (Fig. 1F & 2A). NPM is known to form stable oligomers and localize with the fibrillar center of the nucleolus but also is found in the nucleoplasm and cytoplasm indicating that an additional pool of NPM cycles through different cellular compartments[37]. Surprisingly, we found that the localization of ARF to the nucleolus is exquisitely sensitive to constitutive AKT activity and redistributes ARF to the nucleoplasm in NPM-WT expressing cells, whereas ARF remains nucleolar and stable in the presence of NPM-S48A (Fig. 2C and 2D). To further validate this as an oncogenically driven event, we co-expressed K-Ras^V12^ to constitutively activate endogenous AKT and see a similar Ser48 dependent ARF localization (Fig. S2A). Taken together these results suggest that nucleolar localization of both ARF and NPM are disrupted by AKT phosphorylation of NPM-Ser48, but that a stable pool of NPM persists in oligomeric form. We interpret this as an inability of AKT to target oligomeric NPM due to Ser48 inaccessibility in the pentameric ring, but also that the cycling pool of NPM can be phosphorylated and prevented from forming pentamers, which prevents NPM:ARF sequestration rather than disrupting localization.

**Fig.3:**
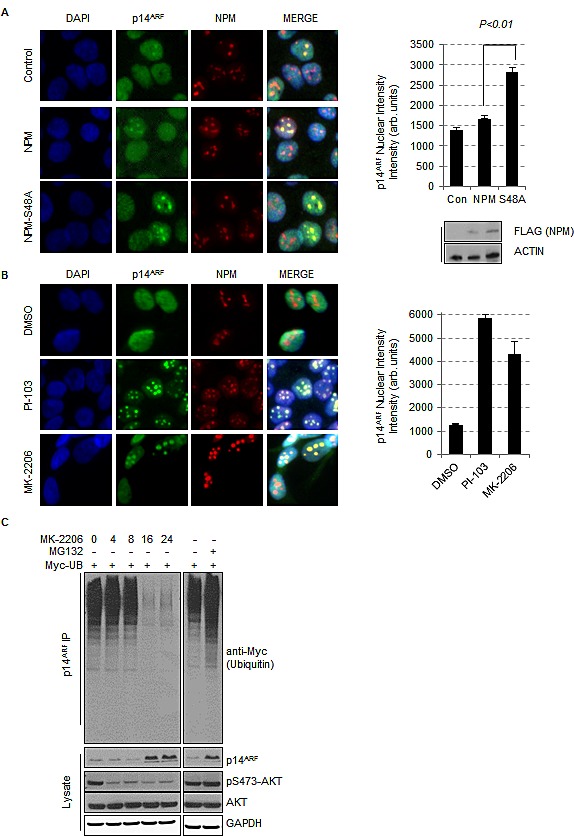
Inhibition of AKT promotes the stabilization and re-localisation of p14ARF to the nucleolus (A) T24 cells were transfected with empty vector (control), FLAG-NPM or FLAG-NPM-S48A or (B) treated with DMSO, PI-103 (0.4 μM), or MK-2206 (5 μM) for 24 hrs. Cells were fixed and stained with anti-NPM (red) and anti-p14ARF (green). Each graph represents the quantification of p14ARF staining intensity in the nucleolus and was performed by In Cell Analyzer 1000 automated epifluorescence microscope. Data are represented as mean ± SEM. (C) Ubiquinitation assay of p14ARF in H1299 cells transfected with Myc-tagged ubiquitin treated with DMSO, MG-132 (10 μM, 16 hrs) or MK-2206 (5 μM) for the times indicated.

In order to investigate whether AKT also regulates ARF localization in human tumor cells we examined ARF localization in T24 cells, a bladder cancer cell line which has elevated ARF expression [35] and constitutive AKT activity due to H-Ras^V12^ mutation. In T24 cells, where NPM is phosphorylated on Ser48 (Fig. 1D), inhibition of AKT decreases the monomeric fraction of NPM (Fig. S2B) and increases NPM oligomers (Fig. S7B). This is also characterized by a change in the localization of phospho-S48-NPM, with the untreated cells showing a nuclear (non-nucleolar) and cytoplasmic localization, that is greatly reduced following AKT inhibition (Fig. S2C and S8D). In tumor cells with constitutive AKT activity, ARF displays a diffuse nucleoplasmic staining similar to *Npm*^−/−^ MEFs or *Npm*^−/−^ MEF reconstituted with NPM-S48E (Fig. 3A and 3B). Exogenous expression of NPM-S48A promotes ARF nucleolar foci and this is not observed with NPM-WT (Fig. 3A), due to endogenous AKT activity. To confirm that AKT was responsible for regulating ARF localization, we next inhibited AKT activity with PI-103 or the pan-AKT inhibitor MK-2206. Both inhibitors promoted almost complete relocalization of endogenous ARF to the nucleoli (Fig. 3B).

We additionally depleted AKT by siRNA and observed ARF nucleolar foci in an analogous manner to treatment with PI-103 or MK-2206 (Fig. S3A). Despite a more pronounced effect of myr-AKT1 on NPM oligomerization (Fig. 1F), depletion of AKT1 or AKT2 appear to regulate ARF foci formation to a similar extent. This suggests that there may be functional redundancy between AKT isoforms towards NPM-Ser48, at least in T24 cells, or that AKT2 may be involved in the stabilization of AKT1 protein levels (Fig. S3A, western blots). Furthermore, the re-localization of ARF to the nucleolus following inhibition of AKT with MK-2206 is characteristically observed where AKT is active, e.g. RAS^V12^ mutated cell lines such as H1299 (Fig. S3B).

NPM mediated sequestration of ARF to the nucleolus is reported to increase ARF protein stability by preventing ubiquitin mediated degradation [38, 40, 43, 44, 57]. We therefore addressed whether ubiquitination of ARF was affected by inhibition of AKT. Following inhibition of AKT, sequestration of ARF to the nucleolus correlated with enhanced co-migration of ARF with oligomeric NPM in semi-native gel electrophoresis (Fig. S2B), reduced ARF ubiquitination and higher ARF levels in whole cell lysates (Fig. S3C). The increased ARF levels following inhibition of AKT are not due to increased transcription as ARF mRNA levels are unchanged following inhibitor treatment (Fig. S3C). Therefore, we can conclude that elevated AKT activity restricts ARF accumulation at the nucleolus.

### AKT promotes the inhibition of MDM2 through a nucleoplasmic NPM/ARF complex

The data presented above argues that phosphorylation of NPM-Ser48 by AKT promotes the nucleoplasmic localization of ARF. The major described function of ARF in cells is as an inhibitor of the E3 ubiquitin ligase MDM2 and consequently leading to increased p53 stability [18, 21, 22]. The NPM mediated sequestration of ARF in the nucleolus has been proposed to increase MDM2 activity in the nucleoplasm[41] and furthermore, relocalization of ARF from the nucleolus to the nucleoplasm is associated with the increased formation of ARF/MDM2 complexes [45]. In T24 cells MDM2 is predominately detected in the nucleoplasm (Fig. S3D & S3E), in agreement with previous reports [47]. Although we observed an increase in ARF nucleolar localization following AKT inhibition, we did not detect any alteration in the cellular distribution of MDM2 (Fig. S3E) suggesting that although NPM and ARF traffic to the nucleolus following inhibition of AKT, MDM2 does not. MDM2 is known to be targeted to the nucleus in response to direct AKT phosphorylation of Ser166 and Ser186, explaining the nuclear localization that we observe in the Ras^V12^ mutant T24 cells (Fig. S3D, S3E & S3F). However, expression of phospho-mimetic MDM2-S166D:S186D (MDM2-DD), that constitutively localizes in the nucleus of T24 cells, shows a similar MDM2 distribution and did not influence the nucleolar localization pattern of ARF upon AKT inhibition (Fig. S3F). In line with previous studies, we observe MDM2 activity is positively regulated by AKT but we find that this is influenced by the presence or absence of ARF (Fig. S3G). Taken together this suggests that NPM constitutively associates with ARF and cycles between a heterodimer organization in the nucleus and an oligomeric state that associates with the nucleolus. We can also assume that AKT promotes MDM2 nuclear localisation, but that its activity is controlled by the AKT mediated NPM-ARF nucleoplasmic pool once there.

In order to investigate whether a NPM/ARF nucleoplasmic complex is responsible for inhibition of MDM2, we performed large-scale stepwise purification of ARF from HeLa nuclear lysates and identified a high molecular weight complex that elutes after consecutive fractionations (ion exchange and size exclusion separation columns) containing both NPM and MDM2 in a highly purified protein fraction, indicating a strong molecular interaction between the constituents of the complex (lower MonoQ fractions A11 & A12, Fig. S4A). As AKT inhibition does not appear to influence the nucleoplasmic/nucleolar distribution of MDM2 (Fig. S3D and S3E), this would suggest that MDM2 is not retained in the NPM/ARF complex upon oligomerisation and therefore cannot stably associate with ARF positive nucleolar foci. This implies that AKT phosphorylation does not influence the ability of nucleoplasmic (monomeric) NPM to associate with ARF and MDM2 per se, but prevents oligomerisation of NPM which can accommodate ARF but not MDM2.

**Fig.4:**
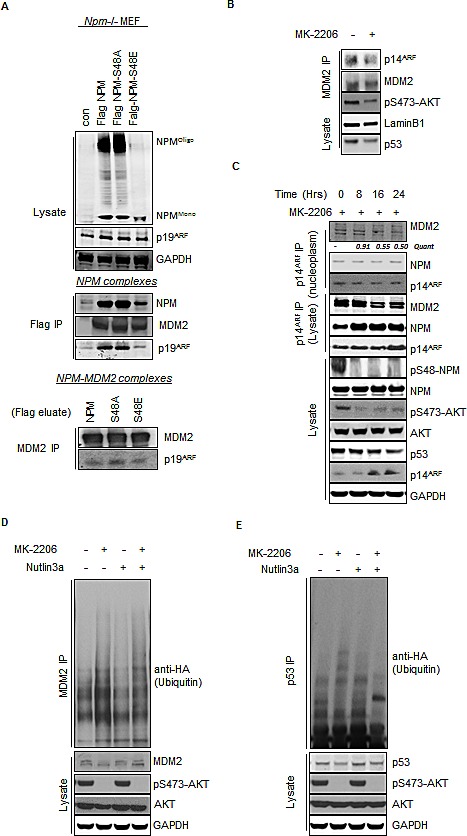
Inhibition of AKT promotes enhanced MDM2 activity via the increased association between NPM and p14ARF (A) *Npm−/−, p53−/−*double null MEF were infected with pBABE retrovirus empty vector and pBABE expressing FLAG-tagged-NPM-WT, NPM-S48A or S48E as indicated. Immunopurification of NPM was done by pulling down with the Flag tag (middle panel) followed by elution of complexes by the Flag peptide and subsequent immunopurification of endogenous MDM2 (lower panel). (B) Nuclear immunoprecipitates of MDM2 from T24 cells treated with MK-2206 (5 μM, 24 hrs). Immunoprecipitates and lysates were blotted with the indicated antibodies. (C) T24 cells were treated with MK-2206 (5 μM) as indicated. p14ARF was immunoprecipitated from whole cell lysates and nuclear extracts and the association with NPM and MDM2 determined by western blot. Immunoprecipitates and lysates were blotted with the indicated antibodies. (D) MDM2 and (E) p53 ubiquinitation assay in H1299 cells transfected with wild type p53, HA-tagged ubiquitin and treated for 16 hrs with DMSO, MK-2206 (5 μM) or Nutlin3A (5 μM) as indicated. Immunoprecipitates and whole cell lysates were probed with the indicated antibodies.

To first confirm that a tripartite NPM/ARF/MDM2 complex exists in the soluble non-nucleolar fraction, a two-step co-immunoprecipitation was performed of FLAG labelled NPM-WT, S48A and S48E from Npm^−/−^ MEFs lysates. NPM complexes were eluted using FLAG peptide and subsequent NPM-MDM2 complexes isolated by immunoprecipitation of endogenous MDM2 from the FLAG elute. The presence of ARF in NPM-MDM2 complex pool reveals that a tripartite complex exists and, although differential stability of the Flag-NPM derivatives make comparisons of levels difficult, this appears independent of the AKT mediated phosphorylation (Fig. 4A). Next we wanted to determine whether the effect of AKT phosphorylation on NPM oligomer formation and ARF localisation affects the ability of NPM-ARF to associate with MDM2. In T24 cells we find an endogenous association of MDM2 with ARF (Fig. 4B and 4C) and also correlates with the low levels of basal MDM2 auto-ubiquitin ligase activity (Fig. 4D). Upon inhibition of AKT with either PI-103 or MK-2206 we observe that the composition of the ARF complexes shifts to exclude MDM2 with a concomitant increase in the apparent association of NPM with ARF due to higher stability (Fig. 4C and S4B). Loss of MDM2 from the NPM/ARF complex was concurrent with incorporation of NPM into oligomers within the nucleolus and accumulation of ARF nucleolar foci (Fig. 3B, 4B and 4C).

As mentioned above, basal MDM2 activity is restricted in T24 cells and as such we observe low background auto-activity or targeting of p53 (Fig. 4D and 4E). Inhibition of AKT and, associated restriction of ARF to the nucleolus, is associated with increased MDM2 auto-ubiquitination and MDM2 mediated (Nutlin3A sensitive) substrate targeting of wild type p53 (Fig. 4D and 4E). Taken together, these results suggest that AKT phosphorylation disrupts NPM oligomerisation and controls MDM2 activity through the restricted compartmentalization of ARF at the nucleolus.

### Inhibition of AKT decreases p53^mut^ stability in a NPM and ARF dependent manner

**Fig.5:**
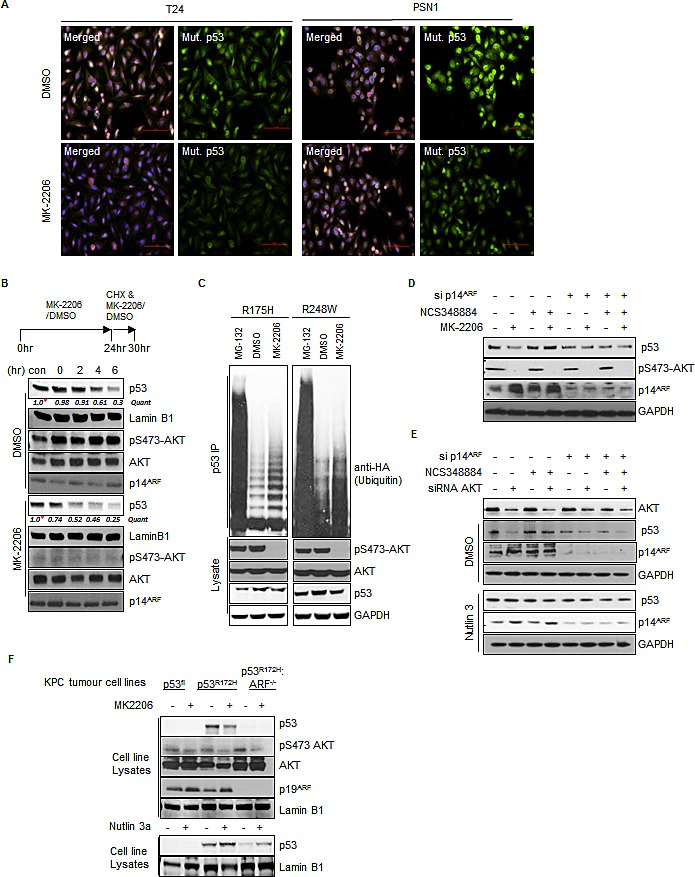
Inhibition of AKT decreases p53mut stability (A) T24 cells and PSN1 cells were treated with MK-2206 (5 μM) or DMSO for 24 hrs. Cells were fixed and stained with DAPI and anti-mutant p53 (OP29 clone). (B) T24 cells were pre-treated with MK-2206 (5 μM) or DMSO for 24 hrs before the addition of fresh media containing cyclohexamide (100 μM) (CHX) in combination with MK-2206 (5 μM) or DMSO for the times indicated. Nuclear extracts were prepared from treated cells and blotted with the indicated antibodies. Quantification is relative to initiation of CHX treatment for both conditions *, MK-2206 is 40% of DMSO control but taken as 1.0 for relative assessment. (C) H1299 cells were transfected with HA-tagged-ubiquitin and mutant p53 (R175H or R248W) as indicated. Transfected cells were treated with DMSO, MK-2206 (5 μM) or Nutlin3A (5 μM) for 16hrs as indicated. p53 immunoprecipitates and whole cell lysates were probed with the indicated antibodies. (D) T24 cells were transfected with non-targeting control or p14ARF siRNA and treated with DMSO, MK-2206 (5 μM) or the NPM oligomerisation inhibitor NCS348884 (4 μM) (Qi et al., 2008) as indicated. (E) T24 cells were transfected with non-targeting control, AKT1, or p14ARF siRNA. Cells were treated with NCS348884 (4 μM), Nutlin3A (5 μM) or DMSO as indicated. Whole cell lysates were probed with the indicated antibodies. (F) KPC mice derived KRAS^G12D^ p53 Floxed (p53^Fl^), KRAS^G12D^ p53^R172H^ ARF+/+ and KRAS^G12D^ p53^R172H^ ARF^−/−^ pancreatic tumor cells were treated with MK-2206 (1μM), Nutlin3A (5μM) or DMSO as indicated. Whole cell lysates were probed with the indicated antibodies.

Since inhibition of AKT promotes ARF localization to the nucleolus and enhanced MDM2 activity, we wished to address what effect increased MDM2 activity has on endogenous p53. Upon inhibition of AKT we observe a decrease in the level of endogenous p53 in T24 cells (Fig. 4B). We were particularly intrigued as T24 cells express p53^mut^ (in-frame deletion of Tyr126). Since MDM2 can degrade p53^mut^ *in-vivo* [27], we hypothesized that constitutive AKT activity may contribute to enhanced p53^mut^ stability via the phospho-S48-NPM dependent localization of ARF to the nucleoplasm and subsequent MDM2 inhibition. Immunofluorescence using a specific antibody (OP-29) that only recognises p53^mut^ in the native conformation revealed a substantial decrease in p53^mut^ staining following inhibition of AKT in T24 and PSN1 cells (RasV12; p53^mut^) (Fig. 5A). The reduction of p53 expression following AKT inhibition is not transcriptionally mediated as the levels of p53 mRNA do not significantly change following inhibitor treatment (Fig. S3C). In order to confirm that inhibition of AKT was directly modifying p53^mut^ turnover, we examined p53^mut^ stability following the addition of cyclohexamide in cells that were pretreated with or without MK-2206 for 24 hrs (to allow ARF nucleolar localization). In MK-2206 treated cells, the initial level of p53^mut^ is lower compared to controls (Quant ratio = 0.4,* in Fig. 5B) and the half-life of p53^mut^ is reduced compared to controls, indicating that p53^mut^ turnover is accelerated following inhibition of AKT (Fig. 5B). Similar results were also obtained using S^35^ Met/Cys pulse chase analysis (Fig. S5A). Furthermore, the effects of AKT inhibition appear to be specific to AKT and not due to downstream signaling targets as we do not observe any alterations to p53^mut^ stability following treatment with the m-TOR inhibitor CC1-229 (Fig. S5B). In order to extend these observations to other p53^mut^ variants, we examined the ubiquitination of two p53 mutants with common hotspot mutations (R175H & R248W). Following AKT inhibition the ubiquitination of both p53^R175H^ and p53^R248W^ is enhanced, indicative of increased MDM2 activity (Fig. 5C).

AKT has been reported to phosphorylate and activate MDM2 leading to de-stabilization of p53 [58, 59]. Similarly we find that inhibition of AKT in ARF null MCF7 cells leads to the stabilization of p53 (Fig. S3G). However, re-expression of ARF appears to be dominant over AKT mediated control of MDM2 activity (Fig. S3G). To assess if inhibition of AKT and regulation of ARF localization represented a general mechanism of regulating p53^mut^ stability we examined p53 expression in a range of cell lines from different histopathological origins, with wild type and p53 mutations, following treatment with PI-103. Short term treatment with PI-103 reduces the levels of phospho-S48-NPM and is accompanied with a rapid decrease of p53 levels in SQ20B (Fig. S5C). After 16 hr exposure to PI-103 a reduction in p53 stability is seen in all cells except *ARF* null A549, PANC1 and *ARF* methylated Lovo cells (Fig. S5C and 6D).

In order to confirm that NPM oligomerisation was regulating p53^mut^ stability we reasoned that direct perturbation of NPM oligomers should therefore regulate p53^mut^ stability in an analogous manner to phosphorylation by AKT. NSC348884 is a compound that directly prevents formation of NPM oligomers [60] and treatment with this compound abrogated the effects of AKT inhibition or siRNA mediated knockdown of AKT on p53^mut^ levels (Fig. 5D and 5E). Moreover, the stability of p53 was reduced by siARF and promoted by the MDM2 inhibitor Nutlin3A independently of NSC34884 (Fig. 5E). Conversely, *Npm*^−/−^:*p53*^−/−^ MEFs transfected with NPM-WT and p53^R248H^ display increased stability of p53 upon expression Myr-AKT1 (Fig. S5D). On the other hand, basal p53^R248H^ levels appear more stable in the context of NPM-S48A, and importantly are not sensitive to myr-AKT (Fig. S5D). These findings suggest that disruption of NPM quaternary structure is sufficient to stabilize p53^mut^ and functions upstream of both ARF and MDM2.

To ultimately confirm a positive role for AKT on p53^mut^ stability we took advantage of pancreatic tumor cell-lines derived from the KRas^G12^ Pdx1-cre, p53^R172H^ (KPC) mouse models of pancreatic ductal adenocarcinoma. We derived tumor cells from KPC (KRas^G12D^: p53^R172H^), *Trp53*flox (KRas^G12D^: p53^fl^), and ARF−/− (KRas^G12D^: p53^R172H^:ARF^−/−^) mice which have been described previously [21, 29, 61-63]. The individual cell lines show similar growth and survival metrics (Fig. S5E) but p53^mut^ protein is clearly only stabilized in the presence of ARF (Fig. 5F). Furthermore, our results indicate that p53^mut^ is degraded by MDM2 as Nutlin3A treatment stabilizes protein levels both in the presence and absence of ARF (Fig. 5F, lower panels), implying that constitutive MDM2 activity in the absence of ARF keeps p53^mut^ levels low.

### Inhibition of AKT relieves the p53^mut^ suppression of p73

**Fig.6:**
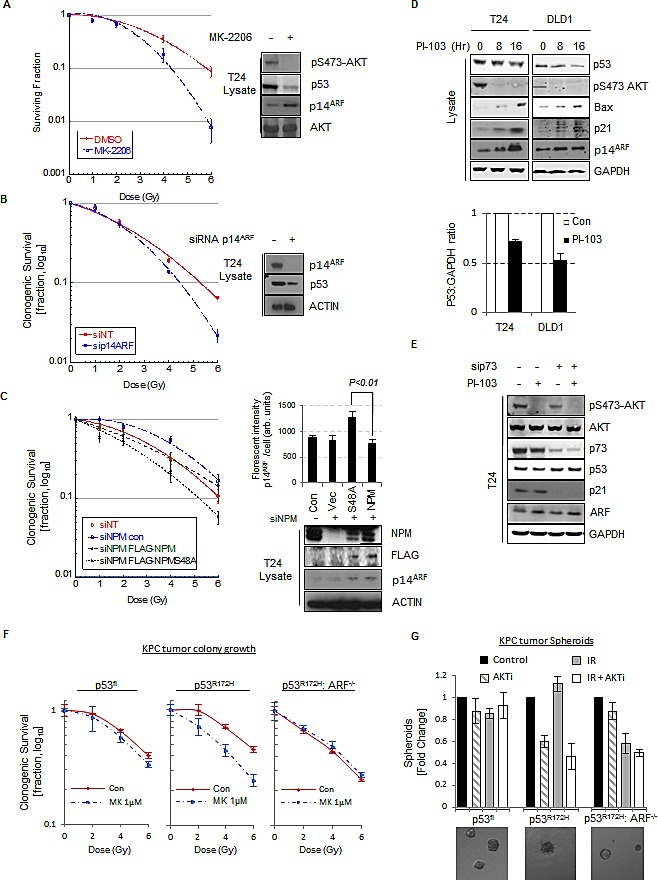
p14ARF has oncogenic activity in p53mut cells (A) Clonogenic survival of T24 cells following treatment with ionizing radiation at the indicated doses. Cells were pre-treated with MK-2206 (5 μM) or DMSO before irradiation. Whole cell lysates were blotted with the indicated antibodies. (B) As in (A) except cells were transfected with non-targeting (NT) control or p14ARF siRNA before irradiation. Whole cell lysates were blotted with the indicated antibodies. (C) As in (A) except T24 cells were transfected with NT or NPM siRNA and siRNA resistant FLAG-NPMWT or FLAG-NPM-S48A as indicated. Whole cell lysates were blotted with the indicated antibodies. Right, quantification of p14ARF nuclear fluorescence by In Cell Analyser 1000 automated epifluorescence microscope. Data are represented as mean ± SEM. (D) T24 cells (p53Mut) and DLD1 cells (p53Mut) were treated with PI-103 (0.4 μM) for the indicated times. Bars indicate relative level of p53 at 8 Hr. Whole cell lysates were blotted with the indicated antibodies.(E) T24 cells transfected with NT or p73 siRNA against p73 were treated with PI-103 (0.4 μM) for 16 hrs. Whole cell lysates were blotted with the indicated antibodies. (F) Clonogenic survival of KPC mice derived KRAS^G12D^ p53 Floxed (p53^Fl^), KRAS^G12D^ p53^R172H^ ARF+/+ and KRAS^G12D^ p53^R172H^ ARF−/− pancreatic tumor cells following treatment with radiation at the indicated doses. Cells were pre-treated with MK-2206 (1μM) or DMSO before irradiation. (G) Bars showing 3D clonogenic survival of KPC mice derived KRAS^G12D^ p53^Fl^, KRAS^G12D^ p53^R172H^ ARF+/+ and KRAS^G12D^ p53^R172H^ ARF−/− pancreatic tumor cells following treatment with 6Gy radiation. Cells were pre-treated with MK-2206 (1μM) before radiation. Lower panel shows representative images of 3D colonies.

The data presented above suggests that inhibition of AKT promotes increased turnover of p53^mut^. Inhibiting p53^mut^ function is of particular interest as mutations in p53 are attributed with gain of function phenotypes that accelerate tumor development, promote increased metastasis and resistance to therapy [24, 25]. Mutations in p53 mediate resistance to DNA damage induced by ionizing radiation (IR) [64] and therefore if our model is correct, AKT inhibition should revert this effect. Upon treatment with PI-103, the initial high levels of p53^mut^ protein in T24 and SQ20B cell lines are reduced concomitantly with decreased phospho-S48-NPM and increased sensitivity to IR (Fig. S6A and S6B) in line with previous reports [65]. Moreover reduction of p53^mut^ independently of AKT inhibition, via siRNA mediated silencing of p53, increases the sensitivity of T24 cells to IR (Fig. S6C). Inhibition of AKT with MK-2206 mediates reduction in p53^mut^ and clonogenic survival upon exposure to IR (Fig. 6A), suggesting that the mechanistic regulation of NPM dynamics and ARF localization correlates with therapeutic resistance to DNA damage. Furthermore siRNA mediated knockdown of ARF expression similarly increased the sensitivity of T24 cells to IR, indicating that ARF promotes resistance to IR in p53^mut^ T24 cells (Fig. 6B). Nucleoplasmic ARF is targeted for degradation by the E3-ligase ULF [57], therefore manipulation of ULF levels should have the opposite effect on sensitivity to IR. Indeed, depletion of ULF resulted in elevated ARF levels and enhanced resistance of T24 cells to IR in an ARF dependent manner (Fig. S6D). To confirm that phospho-S48-NPM is responsible for IR resistance, we next ablated NPM expression in T24 cells and expressed siRNA resistant NPM and NPM-S48A mutants. In agreement with our model, overexpression of NPM-S48A increases ARF nucleolar foci (Fig. 6C, bars) and decreases clonogenic survival (Fig. 6C). These results suggest that AKT mediated phosphorylation of NPM-Ser48 promotes cellular resistance to IR by promoting ARF relocalization to the nucleoplasm and the stabilization of p53^mut^.

A classical gain of function phenotype associated with p53^mut^ is the inhibition of the p53 family member p73 [24, 25]. As p73 shares many transcriptional targets with p53, activation of p73 following DNA damage can be inhibited by high stable levels of p53^mut^ through dominant negative suppression of classical p53 targets [66, 67]. Therefore we reasoned that the increased sensitivity to IR following reduction in p53^mut^ stability may be due to derepression of p73 function. Intriguingly, treatment of T24 or DLD1 p53^mut^ cell lines with AKT inhibitors, PI-103 or MK-2206 leads to the induction of p53/p73 target genes, p21 and BAX (Fig. 6D, 6E and S6E). Furthermore, the transcription of p21 in T24 cells is p73 dependent as siRNA mediated silencing of p73 expression significantly inhibited p21 expression (Fig. 6E and S6E). The induction of p21 was accompanied with a G1/S cell cycle arrest (Fig. S6F) and the induction of cellular senescence markers (β-galactosidase) (Fig. S6G). Moreover, the induction of senescence following IR was significantly increased in cells that were pre-treated with MK-2206 (Fig. 6G). The observations were further confirmed in the KPC derived pancreatic cancer cells described above. Clonogenic assays show increased sensitivity of the KPC.p53^R172H^ (ARF^+ve^) cells to combination of AKT inhibition and IR, which is not exhibited by the KPC.p53^fl^ or KPC.p53^R172H^:ARF^−/−^ cells (Fig. 6F). Interestingly we also see that tumor 3D spheroid growth of KPC.p53^R172H^ (ARF^+ve^) cells are resistant to IR but become sensitive to treatment following AKT inhibition (Figure 6G), further confirming our hypothesis. Overall the data suggests that inhibition of AKT derepresses p53/p73 target genes which in turn restore the normal cellular response to DNA damage.

### Inhibition of AKT represses p53^mut^ stability *in-vivo* and sensitizes PSN1 xenografts to IR

The data presented suggests that the modulation of NPM quaternary structure through inhibition of AKT regulates ARF localization and p53^mut^ stability in cell-culture assays. This is turn mediates resistance to IR. We therefore wished to extend these observations in order to determine if inhibition of AKT could modulate p53^mut^ stability and responses to IR *in-vivo*. To determine whether phospho-S48-NPM affects p53^mut^ levels *in-vivo*, we stained serial sections of SQ20B xenografts from mice treated with either DMSO or PI-103 and observed decreased phospho-S48-NPM positivity and reduced levels of p53^mut^ in PI-103 treated animals (Fig. S7A). However, overall tumor growth upon treatment with PI-103 was not grossly affected [68]. As p53^mut^ accelerates the development of pancreatic cancer [69], we extended these findings by utilizing a pancreatic PSN1 xenograft model which includes stromal support to appropriately replicate the human disease [70].

**Fig.7:**
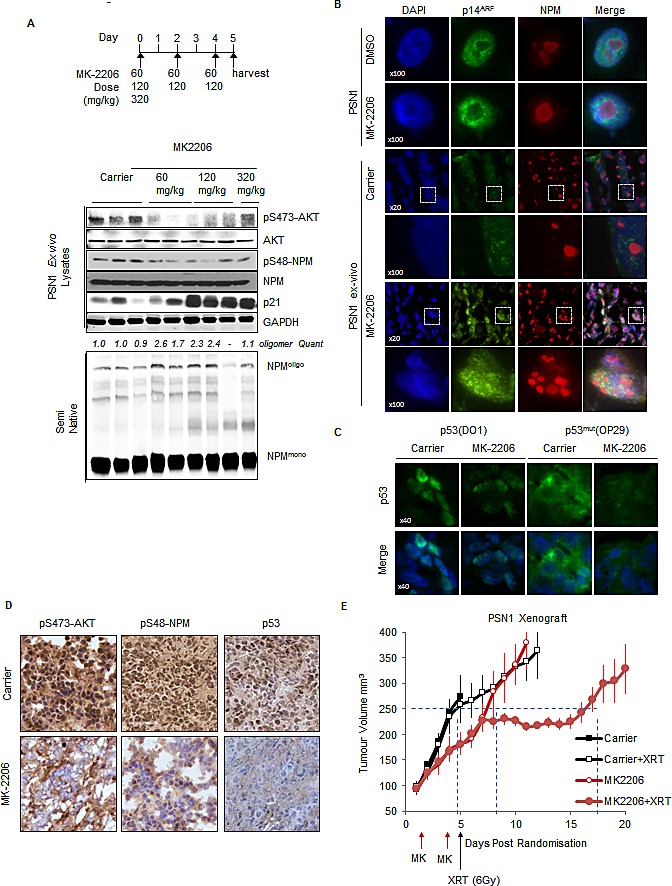
Inhibition of AKT modulates p53 stability *in-vivo* and synergizes with ionizing radiation to inhibit tumor growth (A) PSN1 xenografts (PSN1 cells co-injected with LTC-14 stellate cells) established in the flank of athymic nude mice were treated with MK-2206 (60 mg/kg-320 mg/kg) as indicated or β-cyclo-dextrin (1.5 mg/ml) carrier. Xenograft tumors were lysed and lysates probed by western blot with the indicated antibodies. (B-D) Sections of PSN1 xenografts treated with three consecutive doses of MK-2206 (60 mg/kg). (B) Sections of PSN1 xenografts and *in-vitro* PSN1 cells fixed and stained with anti-NPM (red) and anti-p14ARF (green). (C) PSN1 xenografts treated with MK-2206 or carrier were stained with DAPI, anti-p53 (DO1) or p53mut (OP29 clone) (D) PSN1 xenografts treated with MK-2206 (60 mg/kg) or carrier were stained by immunohistochemical methods with anti-pS473-AKT, anti-phospho-S48-NPM (pS48-NPM) or p53. (E) PSN1 xenografts established in the flank of athymic nude mice were injected subcutaneously with two alternate day doses of MK-2206 (60 mg/kg) or carrier. Mice were subsequently treated with a single dose of IR (6 Gy) and tumor volumes measured regularly with callipers. Dash lines indicate tumor growth differential at 250 mm^3^.

We confirmed that the regulation of NPM oligomerisation and p53^mut^ levels in PSN1 cells were affected by AKT inhibition with MK-2206 *in-vitro* (Fig. 5A, S5B and 7B). PSN1 cells were injected into athymic nude mice which were subsequently treated with MK-2206 or carrier as indicated (Fig. 7A). Tumors were excised and reduced levels of phospho-S473-AKT and phospho-S48-NPM are apparent in the tumor lysates of MK-2206 treated mice, correlating with the enhanced oligomerization of NPM and induction of p21 expression (Fig. 7A). Additionally, we verified that increased p21 expression in MK-2206 treated PSN1 xenografts was linked to inhibition of AKT, decreased phospho-S48-NPM, ARF nucleolar re-localization and decreased p53^mut^ expression by *ex-vivo* staining of tumor sections by immunofluorescence and immunohistochemical staining (IHC) (Fig. 7B, 7C and 7D). In agreement with previous reports [9], PI-3K and AKT inhibitors have little effect on tumor growth as single agents (Fig. S7C). However, our data demonstrates that reduction of p53^mut^ levels reinstates tumor suppressor and DNA damage responses (Fig. 6A, S6A and S6B). We therefore selected the minimal dose that reduced AKT activity (60 mg/kg) for xenograft studies in combination with a single dose of IR (6 Gy, XRT) to determine whether synergy can be achieved *in-vivo*. MK-2006 induced a tumor growth delay of 3.3 days compared to control animals, however, overall survival (time to sacrifice) was not affected. In contrast, the combination of MK-2206 with a single dose of XRT resulted in a growth delay of 12.5 and 9.2 days compared to either XRT or MK-2206 alone (Fig. 7E and S7D). Moreover, this resulted in a significant increase in survival of the treated animals (represented here by the surrogate measure of 4 times tumor volume from time of randomization and initiation of treatment) compared to controls (p<0.0001 Mantel Cox Log-Rank; p<0.0001 Gehan-Breslow-Wilcoxon).

### Phospho-S48-NPM is prevalent in human tumors

**Fig.8:**
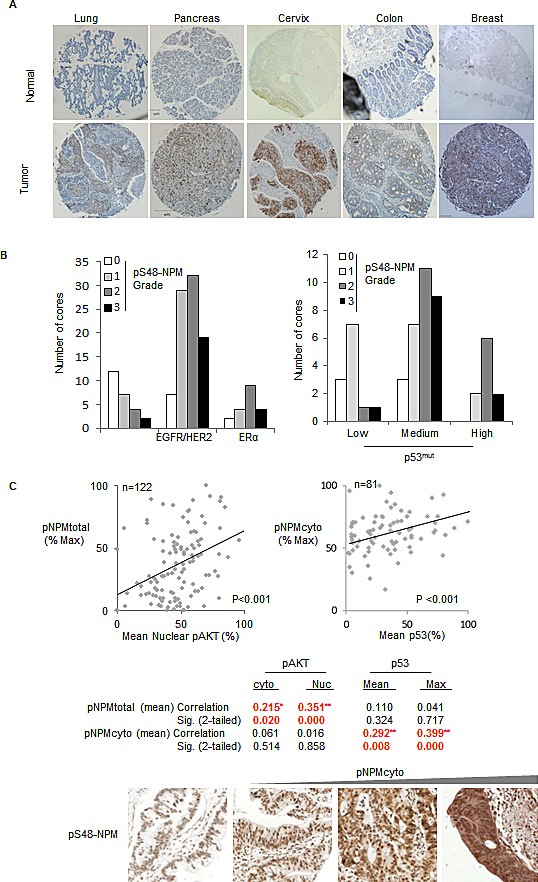
Phospho-S48-NPM correlates with p53 levels in human tumors (A) Tissue micro array sections (US Biomax) of both normal and tumor derived tissue from lung, pancreas, cervix, colon and breast were stained with pS48-NPM. All images are 5 x magnifications and scale bars represent 200 μm. Total numbers of samples analysed by automated Aperio scanning and those with low, medium or high degrees of staining are shown in Table 2. (B) (Left) Data from the breast tumor microarray demonstrating the degree of phospho-S48-NPM staining in those cores annotated as EGFR/HER2 positive (low and high) or estrogen receptor (ER) +ve. (Right) Phospho-S48-NPM staining in cores scored low, medium or high for p53. (C) Scatter plots demonstrating bivariate correlation of automated total pS48-NPM and nuclear phospho-S473-AKT staining in human pancreatic ductal adenocarcinomas (n=122, left), or cytoplasmic pS48-NPM in samples with p53 positivity (n=81, right). Below, representative images for comparison of variation in pS48-NPM^cyto^ scoring of pancreatic ductal adenocarcinoma tumor micro array sections.

Having established that the phosphorylation of NPM-Ser48 by AKT promotes ARF nucleoplasmic localization, MDM2 inhibition and the stabilization of p53^mut^, we next wished to address if phosphorylation of NPM-Ser48 was a common phenomenon, and potentially contributing to the stabilization of p53^mut^ in human tumors. We addressed the levels of phospho-S48-NPM by IHC staining of tumors from tissue samples (pancreas, lung, colon and breast) that frequently activate the EGFR/RAS/PI-3K pathway. In addition, we also included cervical tumors as human papillomavirus (HPV) infected tissue that both overexpresses NPM and display elevated AKT activity [71], suggesting that this tumor material would serve as an additional control for elevated phospho-S48-NPM expression. We detected phospho-S48-NPM in tumor material that was reduced by a blocking peptide and was non-concordant with total NPM (Fig. S8A & S8B). Tissue sections from pancreas, lung, cervix, colon & breast stained positive in greater than 50% cases and were scored as low, medium or high depending on reactivity to the pS48-NPM antibody (Fig. 8A and Table S1). This data together with the cell line analysis (Fig. S5C) suggests that NPM quaternary structure is perturbed in a variety of human tumors. The oncogenic disruption of NPM is likely to disrupt additional signaling pathways which may promote tumorigenesis in p53 null or p53 inactivated tumors (e.g. cervical cancer), [36, 37] but the increased stability of p53^mut^ is increasingly observed to be prognostic in breast cancer [72]. EGFR/HER2^+ve^ and ERα^+ve^ breast cancers have previously been described to have elevated AKT activity [73, 74], we therefore aimed to correlate phospho-S48-NPM staining, with EGFR/HER2 positivity, p53 staining and tumor stage. Interestingly, the highest levels of p53^mut^ segregated with advanced tumor stage and the degree of phospho-S48-NPM staining correlated with EGFR/HER2 positivity and increased p53 staining intensity indicative of p53^mut^ (Fig. 8B and S8C). Not all EGFR/HER2^+ve^ and ERα^+ve^ breast cancers have p53^mut^, however the coalescence of both mutations appears to promote advanced stage disease [75]. Our results suggest that AKT contributes to this effect by stabilizing p53^mut^ protein, however, previous reports indicate that AKT reduces p53 levels via nuclear targeting of MDM2. To confirm whether these correlations are due to the presence of ARF we examined AKT activity and p53 levels in a large cohort of invasive breast cancer where ARF expression is known (The Cancer Genome Atlas, TCGA, http://cancergenome.nih.gov/). We find that p53 protein levels negatively correlate with increased AKT activity, but only in tumors where the *CDKN2A* transcript is detectable above background (values in green, Table S2). Importantly the negative correlation is lost in tumors where *CDKN2A* is expressed (therefore likely to be ARF positive) or when mutations in *TP53* have been annotated (Table S2). To robustly test AKT activity we examined levels of the AKT substrate pRas40, which is used to monitor AKT activity in patients treated with MK-2206 [10]. We first confirmed that AKT activity correlates with substrate phosphorylation, and found AKT substrate activity has an overall positive correlation with total p53 levels (values in red,Table S2). Furthermore, we find that this positive correlation can be found for both p53^WT^ and p53^mut^ tumors, but is restricted to cases where *CDKN2A* transcript is detectable above background. Importantly, the positive correlation is lost in the absence of *CDKN2A* message (i.e. ARF^-ve^) (Table S2). This supports previous data on AKT mediated targeting of MDM2 and our current observations, as AKT can promote either negative or positive regulation of p53 depending on the presence of ARF and its ability to restrict MDM2 activity in the nucleoplasm (Fig. S8F).

The pancreatic TMA indicated 50% phospho-S48-NPM positivity despite a propensity for all pancreatic tumors to harbor activating RAS mutations (Fig. 8A). Activating mutations occur at residues G12-, G13- or Q61-KRAS and result in a range of amino acid substitutions that can differ in the effector pathway and/or potency of activation [76]. Not surprisingly, AKT activity has been described to vary in pancreatic tumors [77]. To address the correlations between AKT substrate activity and p53 levels, we obtained a fresh pancreatic TMA and confirmed the correlation between phospho-S48-NPM, p53 and AKT within this cohort (Fig. 8C). We also observe that 50% of the tumors to be in the lowest quartile of phospho-S48-NPM staining and display low AKT, potentially indicating variations in PI3K activation by different mutations of RAS (Fig. 8C, left scatter plot). Importantly, total phospho-S48-NPM staining significantly correlates with active AKT (phospho-S473-AKT), with the highest correlation in the nuclear compartment (Fig. 8C).

Interestingly, the ‘RXXS’ motif incorporating NPM-Ser48 lies within a characterized nuclear export sequence [55] (Fig. 1B) and a proportion of phospho-S48-NPM is cytoplasmic (Fig. 2A, Fig. S2C & S8D). We next restricted scoring to cytoplasmic positivity, solely within pancreatic ductal adenocarcinomas and scored for elevated p53 staining indicative of p53^mut^. Of the total 122 tumors, 81 scored positive for p53 and the degree of p53 staining correlated with the levels of phospho-S48-NPM^cyto^ (<p=0.001) (Fig. 8C). As the *INK4A/ARF* locus can be deleted, methylated or transcriptionally repressed, we obtained mRNA from a subset of tumors for which material was available (40 tumors). We determined that 26/40 pancreatic tumors were positive for ARF mRNA and observed a significant correlation between phospho-S48-NPM and p53 staining intensity in tumors where ARF was expressed (Fig. S8E).

Overall the data suggest that an AKT/NPM/ARF pathway contributes to the maintenance of elevated levels of p53^mut^. Thus, we can present a model (Fig. S8F) whereby NPM and ARF are constitutively associated in high molecular weight complexes at the nucleolus. AKT phosphorylation on Ser48 prevents NPM oligomerization, thereby increasing nucleoplasmic NPM/ARF that forms an inhibitory complex with MDM2 and results in increased p53^mut^ stability.

## DISCUSSION

The data presented in this report describes how the stability and localization of ARF is regulated by NPM quaternary structure. Previous reports have demonstrated that the inhibition of NPM oligomerisation using small molecule inhibitors [60] or RNA aptameres [78] promotes the nucleoplasmic localization of NPM and ARF with accompanying activation of p53, but the physiological context was not known. We have found that AKT limits NPM oligomerisation (Fig. 1), which in turn restricts NPM nucleolar localization and accumulation of ARF at the nucleolus (Fig. 2 & 3). In tumor cells with constitutive activation of AKT and mutation of p53, the promotion of nucleoplasmic ARF/NPM leads to the subsequent stabilization of p53^mut^ through inhibition of MDM2 (Fig. 4 & 5).

Although ARF has long been recognized as an inhibitor of MDM2 [21, 22], debate exists regarding the cellular location and mechanism of ARF mediated MDM2 inhibition. ARF has been reported to promote p53 stability by sequestering MDM2 to the nucleolus [79, 80], whereas others have reported that nucleoplasmic ARF suppresses MDM2 activity [46-48, 81]. Our data argues for a model whereby MDM2 is inhibited by a nucleoplasmic pool of NPM associated ARF (Fig. 4 and 5). Oligomerisation of NPM promotes accumulation of ARF at the nucleolus but appears incompatible with MDM2 association (Fig. S2B and 4). In agreement with this model, overexpression of NPM and sequestration to the nucleolus has been proposed as mechanism whereby tumor cells inactivate and stabilize ARF [41, 48, 57, 81],

NPM has also been reported to bind and stabilize p53 directly [82] and in addition, promote p53 stability by binding MDM2 [83]. Kurki et al. reported that following UV or viral stress, NPM re-localizes to the nucleoplasm from the nucleolus and is associated with MDM2, in an analogous manner to the increased formation of ARF/MDM2 complex [45]. In agreement, failure to re-localize NPM to the nucleoplasm prevents NPM mediated inhibition of MDM2 [84]. Our results suggest that inhibition of MDM2 by NPM and ARF are not independent and respond to cellular stress via disruption of NPM oligomerization.

Although we have focused on the AKT mediated regulation of p53^mut^ stability, the observation that regulation of NPM oligomerisation governs ARF localization may also be a direct mechanism which facilitates p53 stabilization following oncogene activation in the absence of DNA damage [19]. In the absence of additional mutations, oncogene activation leads to p53 stabilization and the induction of cellular senescence. Furthermore, growing evidence suggests that AKT activity can mediate p53 stabilization and induction of senescence [85-87]. AKT is known to directly phosphorylate S166-MDM2 leading to enhanced activity and reduced p53 levels, which in agreement with previous studies where observations were made in cells with *INK4a/ARF* deletions [58, 59], we find AKT inhibition increases p53 levels in ARF null MCF7 cells (Fig. S3G). Importantly, we find that expression of ARF stabilizes p53 but is now subject to NPM mediated control and AKT inhibition decreases p53 levels. This is in line with emerging evidence where constitutive activation of AKT has been demonstrated to inhibit MDM2 activity [85]. Moreover, we find that correlations exist between AKT activity and p53 levels in breast and pancreatic cancer cohorts, and are dependent on *CDKN2A* expression levels (Table S2). This is a relevant point for the administration of AKT inhibitors in the clinic where efficacy is likely to rely on ARF status as well as p53^mut^.

The induction of senescence following oncogene activation has been unequivocally demonstrated to require ARF [19-22]. However, our data and reports by others [41, 46-48, 81] suggest that the regulation of ARF cellular localization is also required for inhibition of MDM2. Recent studies have reported that the strength of signal originating from oncogene activation is also important in the ARF mediated activation of p53 [88-90]. As ARF localization depends on NPM oligomerisation this suggests that the strength of a signal must be sufficient to disrupt the equilibrium and release ARF, thereby creating a cellular sensor of stress or oncogene activation. We have demonstrated that phosphorylation of Ser48 by AKT contributes to this process, but additional residues of the N-terminal of NPM have been predicted *in-silico* to regulate oligomerisation [53]. Therefore, NPM may be able to integrate multiple signals from different kinases in order to control ARF localization.

Approximately 40% of human tumors have a missense mutation in p53 [91], but mutations alone do not account for the inherent stability of p53^mut^ nor the heterogeneity of levels in tumor tissues [27, 29, 30, 92]. Since highly stable p53^mut^ protein is considered oncogenic, it is important to identify the molecular mechanisms that contribute to p53^mut^ stability, as they offer novel therapeutic routes to target tumors.

Growing evidence suggests that since MDM2 can mediate degradation of p53^mut^ in normal tissue [27, 92] the stabilization of p53^mut^ is not due to the lack of transcriptional activation of MDM2. Therefore, the stabilization of p53^mut^ is not due to loss of MDM2 but suppression of MDM2 activity which arises as cells become transformed. Therefore targeted therapies that re-engage MDM2 activity and destabilize p53^mut^ have the potential to sensitize p53^mut^ cells to therapy. In agreement with this approach tumor cells are often addicted to heat sock proteins (HSP) that promote p53^mut^ stability. HSP not only directly stabilize p53^mut^, they also inhibit E3 ligases (MDM2 and CHIP) responsible for p53 ubiquitination. Treatment with the HSP90 inhibitor 17AAG or the HDAC6 inhibitor SAHA, releases MDM2 and p53^mut^ from this HSP “cage” leading to the rapid destabilization of p53^mut^ and the sensitization of p53^mut^ tumor cells to chemotherapeutic agents [31, 32].

Our data indicates that in tumors where NPM is unable to oligomerize, retention of ARF expression contributes to the stabilization of p53^mut^ cells. In these cases, treatment with AKT or PI-3K inhibitors reduces p53^mut^ stability and sensitizes tumor cells to therapies that engage p73. MDM2 activity is absolutely required for this phenomenon as the MDM2 inhibitor Nutlin3A blocks p53^mut^ turnover which is induced following inhibition of AKT (Fig. 5E). Furthermore, in our cohort of pancreatic tumors that retained ARF expression, the level of phospho-S48-NPM significantly correlated with elevated detection of p53^mut^ indicating than in certain tumors AKT and ARF contribute to the stabilization of p53^mut^ (Fig. 8, Table S2).

An interesting aspect of this study is that although ARF is a well characterized tumor suppressor [93] the observation that ARF contributes to the resistance against IR through stabilization of p53^mut^ and inhibition of p73, suggests that in some circumstances ARF behaves as an oncogene. Interestingly other reports have suggested ARF has oncogenic activity than can affect tumor growth in a tissue specific manner [94, 95]. Independent of p53, human prostate tumors with *PTEN* loss show increased ARF expression which correlates with the increased aggressiveness of disease [95]. Furthermore, ARF has been shown to protect tumor cells from metabolic stress by promoting the induction of autophagy [94]. These reports argue that in specific tissues ARF can exert tumor promoting activity that is distinct from regulation of p53. However, our data and recent reports from others argue that an additional aspect of an oncogenic role for ARF is the maintenance of p53^mut^ stability. *Pml* knockout mice on a p53^mut^ background displayed stabilization of p53^mut^ with expression of ARF, suggesting that loss of PML promotes oncogenic activation of ARF which in turn stabilizes p53^mut^ through inhibition of MDM2 [28]. Further evidence for a role in ARF mediated stabilization of p53^mut^ came from a study that demonstrated TGF-β1 induced destabilization of p53^mut^ by inhibiting E2F-1 mediated transcription of ARF [96]. Interestingly, AKT has been demonstrated to increase ARF transcription by relieving BMI1 repression of ARF transcription [97] and therefore provides a potent mechanism to stabilize p53^mut^ via the elevated transcription and nucleoplasmic localization of ARF.

The EGFR/RAS/PI3K/AKT pathway has been widely linked with therapeutic resistance of tumors. Evidence *from in-vitro* and *in-vivo* models suggests that PI3K-AKT activation is associated with decreased sensitivity to several chemotherapeutic agents and radiotherapy [3, 7]. Additionally, clinical reports highlight the route of resistance to EGFR and HER2 targeted agents, Cetuximab and Trastuzumab (Herceptin), as being reversible upon PI3K-AKT inhibition [98, 99]. Our results describe one route of tumor initiation where pathway activation promotes oncogenic activity of p53^mut^. While this mutational cooperation may contribute to progression, it more importantly highlights the patient cohort where AKT inhibitors are most likely to display efficacy.

The acquisition of p53 mutations, rather than deletion, can be a predictor of prognosis and is now included as a hallmark of cancer [24, 100]. Recently, genetic studies in mice have demonstrated that p53^mut^ gain of function activity requires mutant K-Ras, thus confirming a genetic link [101]. We provide evidence for RAS/PI3K/AKT mediated resistance of tumors being dependent on ARF and p53^mut^ status and as such, a potential confounder of the efficacy in clinical trials. Together, our results outline how the genetic route of tumor initiation impacts on therapeutic responses and moreover, provides a patient selection strategy to ensure maximal therapeutic benefit of PI3K-AKT agents currently under clinical investigation.

## MATERIALS AND METHODS

### Cell culture and transfection

HT-1080-SG1 and SG2 tumor cell lines were kindly provided by Eric Stanbridge (Stanford, CA), AKT knockout MEF's were kindly provided by Dr. Birnbaum (University of Pennsylvania PA). *NPM^−/−^, p53^−/−^* and *p53^−/−^* MEFs were kindly provided by Pier Paolo Pandolfi (Harvard, Boston MA). PSN1 cells were kindly provided by Thomas Brunner (University of Oxford) and subsequently genotyped at the DDC laboratories, London, to confirm cell identity. The mutation data reflects the information on the COSMIC Cell Line Project database http://cancer.sanger.ac.uk/cancergenome/projects/cell_lines/. All other cell lines were obtained from the American Type Culture Collection (ATCC). Cells were cultured in DMEM containing 4.5 g/l glucose (Invitrogen) supplemented with 10% fetal bovine serum (Invitrogen), penicillin (100 units/ml), and streptomycin (100 mg/ml). All cultures were maintained at 37°C in water saturated 5% CO_2_ with the exception of MRC5 cells which were cultured at 37°C in 3% O_2_/5% CO_2_. Cells were plated to 80% confluence prior to siRNA transfection. Smartpool siRNA (Dharmacon) against NPM, p14^ARF^ and AKT were used where indicated. Individual siRNA against NPM (ACAAGAAUCCUUCAAGAAA) was used in conjunction with re-expression of NPM constructs. sip73 sequences (GCAAGCAGCCCAUCAAGGA and GAGACGAGGACACGUACUA), sip53 (GACUCCAGUGGUAAUCUAC), siULF (GGUAGUGACUCCACCCAUUUU). Cells were transfected with plasmid or siRNA (50 nM) construct using Lipofectamine 2000 (Invitrogen) or Dharmafect (Dharmacon) for 48 hours prior to drug treatment. Mutant p53 plasmids (R248W & R175H) cloned into pcDNA3 vector backbone were a kind gift from Xin Lu (Ludwig Institute for Cancer Research, University of Oxford).

### KPC mice derived cell lines

*Pdx1-cre, K*r*asG12D, Trp53flox, Trp53R172H* and *ARF−/−* mice have been described previously [21, 29, 61-63]. Mice were kept in conventional animal facilities and experiments were carried out in compliance with UK Home Office guidelines. Genotyping was performed by Transnetyx (Cordova, TN, USA). Animals were monitored until showing symptoms of late stage pancreatic cancer and then sacrificed as per institutional guidelines. Tumor and metastatic burden was assessed by gross pathology and histology.

Tumor tissue for preparation of PDAC cell lines was harvested in DMEM. Tumors were disaggregated by fine mincing with scalpels, and plated in growth media (Dulbecco's modified Eagle medium containing 10% fetal bovine serum and 2mmol/L L-glutamine). Cells were allowed to adhere, washed, grown to confluence and then passaged as normal.

### Chemicals and antibodies

All chemicals were purchased from Sigma Aldrich unless stated otherwise. PI-103 was purchased from Merck. CCI-779 was purchased from Sigma. MK-2206 was purchased from ChemieTek. All inhibitors were dissolved as concentrated stock solutions in DMSO (1 mM) and stored at −80°C. Antibodies, anti-AKT1 (#9272), anti-AKT2 (#2964), anti-AKT1 (#2938) anti-phospho-Ser 473-AKT (#9721), anti-p44/42 MAPK (Erk1/2) (#137F5), anti-phospho-p44/42 MAPK (Erk1/2) (Thr202/Tyr204) (#9106) and anti-phospho-S6 (Ser 235/236) (#91B2) were purchased from Cell Signaling Technologies. Anti-GAPDH (2251-1) and anti-p73 (1636-1) were purchased from Epitomics. Anti-HA tag (05-904) and anti-Myc tag (05-724) were purchased from Millipore. Anti-p14^ARF^ (Ab11048 and Ab49166), anti-nucleophosmin (ab10530), anti-ULF (Ab80645), anti-Fibrillarin (Ab5821), anti-Lamin B1 (Ab16048) and anti-p53 (D01 clone) (Ab80645), were purchased from Abcam. Anti-p19^ARF^ (NB-200-174) and anti-p14^ARF^ (NB-200-111) were purchased from Novus Biologicals. Anti-FLAG (M2) was purchased from Agilent Technologies. Anti-actin (A4700) was purchased from Sigma. Anti-p21 (sc6246), anti-MDM2 (SMP14 (Sc-965)), anti-HSP-90 (Sc-69703) and anti-Bax (2D2) were purchased from Santa Cruz Biotechnology. The anti phospho-Ser48-NPM was raised against a synthetic peptide spanning residues 45-56 of NPM (RTVSLGAGAKDE) incorporating phospho Ser (underlined) at position 48. Peptide synthesis and immunizations were carried out by Eurogentec. The anti phospho-Ser48-NPM used in this study was affinity purified against the phosphopeptide. Secondary antibodies anti-mouse and anti-rabbit HRP conjugates were purchased from Pierce and the Jackson Laboratory. Fluorescent tagged antibodies for use on the Licor Odyssey western blot imaging system were purchased from Licor and Invitrogen. Fluorescent conjugated secondary antibodies for immunofluorescence were purchased from Invitrogen.

### Protein biochemistry

### Preparation of cell lysates and western blot

Unless specified otherwise, whole cell lysates were prepared by lysing cells with 1% NP-40 lysis buffer (50 mM Tris-HCl, 150 mM NaCl, 2 mM EGTA, 5 mM MgCl_2_, 1% (v/v) NP-40, 10 mM sodium β-glycerophosphate, 50 mM NaF, 1 mM Na_3_VO_4_, 5 mM sodium pyrophosphate and ‘Complete’ proteinase inhibitor cocktail EDTA free (1 tablet/10 ml lysis buffer (Roche)). Lysates were rotated end over end for 30 min at 4ºC and centrifuged (20,817 × g, 10 min) before the addition of NuPage sample buffer or SDS-PAGE sample buffer (1x concentration, 62.5 mM Tris-HCl pH 6.8, 25% (v/v) glycerol, 2% (w/v) SDS, 0.01% (w/v) bromophenol blue). For immunoprecipitation, lysates were pre-cleared (4 ºC, 1 Hr) with protein-G coupled to magnetic beads (Millipore), prior to incubation with antibody conjugated to protein-G magnetic beads. Lysates and antibody coupled beads were rotated end over end at 4°C for at least 3 Hrs. Immunoprecipitates were washed (4 × 1ml) with lysis buffer minus the protease and phosphatase inhibitors. Immunoprecipitated proteins were boiled in SDS-PAGE sample buffer for western blot analysis.

Nuclear lysates were prepared as described previously [102] with additional modifications. Briefly, 3-5 × 10^6^ cells were trypsinised and harvested by centrifugation (500 × g, 5 min), washed twice in TBS and re-suspended in 1-2 ml ice cold buffer A (10 mM HEPES pH 7.9, 10 mM KCl, 0.1 mM EDTA, 0.1 mM EGTA, 1 mM DTT, 0.5 mM PMSF) by gently pipetting in a 1 ml tip. The cells were left on ice for 15 min to swell, after which 75 μl of 10% NP-40 was added. The tube was vigorously vortexed for 10 sec and centrifuged at 500 × g for 2 min. The supernatant, which constitutes the cytoplasmic fraction, was removed. The nuclear pellet was re-suspended in 150 μl ice-cold lysis buffer (150 mM NaCl, 20 mM Hepes pH 7.5, 0.5 mM EDTA, 0.5% (v/v) NP-40, 10 mM sodium β-glycerophosphate, 50 mM NaF, 1 mM Na_3_VO_4_, 5 mM sodium pyrophosphate and ‘Complete’ proteinase inhibitor cocktail EDTA free (1 tablet/10 ml lysis buffer) and the sample sonicated. The nuclear extract was centrifuged (20,817 × g, 15 min, 4°C) and the supernatant containing the nuclear extract was used as an in-put for immunoprecipitation or added to SDS-PAGE sample buffer as described above.

For western blot, samples were boiled (100 ºC, 5 min) and proteins resolved on NuPage Bis-Tris gels (Invitrogen). Resolved proteins were transferred by western blot to PVDF (Millipore) or Nitrocellulose (Biorad) membrane and blocked in either 5% (w/v) non-fat milk or 5% (w/v) BSA dissolved in PBS, 0.1% (v/v) Tween-20 prior to antibody addition. Those membranes probed for phosphorylated proteins were blocked in 5% (w/v) BSA, TBS (50 mM Tris-HCl, pH 7.5, 150 mM NaCl), 0.1% (v/v) Tween-20. Primary antibody detection was achieved with HRP conjugated secondary antibodies (Pierce) and exposure to X-Ray film (Kodak). For blots which were quantified, samples were run by western blot and transferred onto Nitrocellulose membrane (Biorad). Membranes were blocked in Licor blocking buffer and incubated with primary antibody overnight followed by incubation with fluorescently conjugated secondary antibodies. Membranes were scanned on the Licor Odyssey infrared scanner and signal intensity determined using the Odyssey software (V3.0). Signals were normalized to GAPDH as a loading control. All quantification was done on the same nitrocellulose membrane without stripping.

### *In-Vitro* Kinase assay

Endogenous AKT was immunoprecipitated from T24 cells, glycine eluted and combined with immunoprecipitated HA-NPM, HA-NPM-S48A or anti HA-IP from non-transfected cells as indicated in 1 × kinase buffer (Cell Signaling). The reaction was incubated with cold ATP (20 μM) and radio-labeled gamma ^32^P ATP (2 μM) at 37°C. The reaction was heat inactivated in the presence of denaturing SDS-PAGE sample buffer, separated by SDS-PAGE, western blotted and exposed to a phosphor screen. The membrane was additionally probed by standard western blot.

### Ubiquinitation assays

H1299 cells (1 × 10^6^) were transfected with pcDNA3 expressing either HA-tagged Ubiquitin or Myc-tagged Ubiquitin (10 μg) alone or in combination with pcDNA3 expressing p53^Wt^ (10 μg) or the p53 mutants R175H or R248W (10 μg) using Lipofectamine 2000 (Invitrogen). 24 hrs following transfection cells were treated with 10 μM MG-132, 5 μM MK-2206 or 5 μM Nutlin3A as indicated in the figure legends. Cells were washed twice with PBS and lysed by scrapping in ice cold RIPA buffer (50 mM Tris pH 8.0, 150 mM NaCl, 1% (v/v) Triton X 100, 0.5% (w/v) sodium deoxycholate, 0.1% (w/v) SDS, 1 mM EDTA, 1 mM EGTA, 50 mM NaF, 1 mM Na_3_V0_4_, 10 mM sodium β-glycerophosphate, 5 mM sodium pyrophosphate and ‘Complete’ proteinase inhibitor cocktail EDTA Free (1 tablet / 10ml lysis buffer) supplemented with 1 mM N-Ethylmaleimide. Lysates were rotated end over end (4ºC, 30 min), sonicated, centrifuged (20,817x g, 15 min, 4ºC) and pre-cleared with protein G coupled to magnetic beads (Millipore). Pre-cleared lysates were incubated with the appropriate antibody conjugated to protein G for 3 Hrs. Protein G beads were washed with 50 mM Tris pH 8.0, 150 mM NaCl, 1% (v/v) Triton-X-100, 0.5% (w/v) sodium deoxycholate, 0.1% (w/v) SDS before boiling in SDS-PAGE sample buffer. Samples were resolved on 10% NuPage Bis-Tris gels or 4-8% Nupage Tris-Acetate gels (Invitrogen).

### Semi-native Gel Electrophoresis

Cells were washed twice with ice cold PBS and lysed by scrapping in ice cold lysis buffer (50 mM Tris pH 8.0, 150 mM NaCl, 1% (v/v) Triton X 100, 1 mM EDTA, 1 mM EGTA, 50 mM NaF, 1 mM Na_3_V0_4_, 10 mM sodium β-glycerophosphate, 5 mM sodium pyrophosphate and ‘Complete’ proteinase inhibitor cocktail EDTA Free (1 tablet / 10ml lysis buffer). Lysates were rotated end over end (4ºC, 30 min), centrifuged (21000 × g. 15 min), diluted in the appropriate volume of NuPage sample buffer (samples were not boiled) and immediately loaded onto 10% Bis-Tris Nupage gels or Native Nupage gels. Gels were run at a constant voltage (100 V) at 4ºC before transfer to Nitrocellulose membrane.

### Purification of p14^ARF^ from HeLa Nuclear extracts

Fractionation of HeLa cells was performed as previously described [103]. Briefly, 20 grams of HeLa cell pellets (Cilbiotech, Mons, Belgium) were resuspended in 40 ml lysis buffer (20 mM Tris–HCl pH 7.4, 2.5 mM MgCl_2_, 0.5% (v/v) Nonidet P-40, 1 mM PMSF, 1 mM DTT and 1 mg/ml each of aprotinin, pepstatin, chymostatin and leupeptin) and incubated on ice for 10 min prior to centrifugation (1,300 × g, 4 min at 4°C). The supernatant containing cytoplasmic proteins was discarded and the remaining cell pellet was further resuspended in 40 ml buffer containing 100 mM phosphate, pH 8.0, 0.5 M KCl, 5 mM MgCl_2_, 1 mM EDTA, 0.75% (v/v) Triton X-100, 10% (v/v) glycerol, 1 mM PMSF, 1 mM DTT and 1 mg/ml each of aprotinin, pepstatin, chymostatin and leupeptin and the supernatant containing nuclear proteins was collected. The obtained extract was dialyzed against Buffer A (50 mM Tris-HCl, pH 8.0, 1 mM EDTA, 5% (v/v) glycerol, 1 mM DTT and 0.1 mM PMSF) containing 50 mM KCl. The extract was applied to a 20 ml HiLoad Q Sepharose column (GE Healthcare). The column was washed in Buffer A containing 300 mM KCl and proteins bound to the column were eluted using a linear gradient of 300-700 mM KCl. Fractions containing p14^ARF^ (fractions B8-B2) were pooled, concentrated using Amicon Ultra Ultracel-3K filter units (Millipore) and separated on a Superdex 200 HR 10/30 column (GE Healthcare) in Buffer A containing 150 mM KCl. Fractions containing p14^ARF^ were eluted as two separate pools of different molecular weight (pool I is represented by fractions B6-C1, pool II consists of fractions C4-C10) were pooled, the fractions in each pool were combined and further loaded separately onto a 1 ml MonoQ column (GE Healthcare) in Buffer A containing 150 mM KCl. The column was washed and bound proteins were eluted as described above for the HiLoad Q Sepharose column purification step. At each purification step, aliquots of the obtained fractions were analyzed by western blot for the presence of p14^ARF^ using a specific antibody (Bethyl Laboratories, A300-340A). Fractions identified as containing p14^ARF^ were pooled for the next chromatography step.

### 35S Met/Cys Pulse Chase

T24 cells (1 × 10^6^/10 cm dish) were plated 24 hrs before addition of MK-2206 (5 μM) or DMSO control and incubated for 18 Hrs. MK-2206 or DMSO was maintained in culture media throughout the experiment Cells were washed twice with Met/Cys free media (Invitrogen) and cultured for 1 hr at 37ºC with Met/Cys free media. Following depletion of intracellular Met/Cys stores, cells were incubated with Met/Cys free media supplemented with 200 μCi ^35^S Met/Cys (EasyTag™ EXPRESS35S Protein Labeling Mix 35S, PerkinElmer). Cells were metabolically labeled for 1 Hr before being washed twice with complete media containing unlabeled Met/Cys. Cells were chased for 30 min- 4 Hrs in complete media at 37ºC before being washed twice with PBS and lysed by scrapping in 1% (v/v) NP-40 lysis buffer. p53 was immunoprecipitated as outlined above. Samples were run on a 10% NuPage Bis Tris gel. Gels were dried before exposure to a phosphor screen. Additionally samples were probed by western blot.

### Immunofluorescence and Immunohistochemistry

For analysis of cells using the In Cell Analyzer 1000, automated epifluorescence microscope (GE Healthcare), cells were plated into 96-well plates at a density of 10,000 cells/well and incubated overnight at 37°C with 5% CO_2_ in a humidified incubator. Cells were exposed to inhibitor 24 hours prior to fixation. Cells were fixed with 4% paraformaldehyde and permeabilized with 1% (v/v) TritonX-100 and blocked with a 1 (w/v)% solution of BSA in PBS. Cells were incubated with primary antibody as indicated (1:1000 dilution), overnight at 4°C. Primary antibody was detected using Alexafluor conjugate secondary antibodies (Invitrogen) at 1:1000 dilution. Cells were counterstained with DAPI (1 μg/ml). Foci were detected using an In Cell Analyzer 1000 automated epifluorescence microscope (GE Healthcare). For all other immunofluorescence based experiments cells were grown on coverslips, fixed with 4% (v/v) PFA and permeabilized with 0.1% (v/v) Triton X-100. Coverslips were blocked with 3% (w/v) BSA dissolved in PBS and incubated with primary antibody (1/100) prepared in blocking buffer, overnight at 4ºC. Coverslips were washed with PBS and incubated with the appropriate fluorescently conjugated secondary antibody (1/500) for 1 Hr at room temperature. Coverslips were washed (3 X PBS) and images captured using a Nikon 90i epifluorescent microscope or LSM 710 (Zeiss) confocal microscope.

For frozen tissue sections, slides were fixed in acetone for 10 minutes at room temperature. Slides were dried, washed in PBS and non-specific binding blocked with 3% (v/v) normal bovine serum (NBS), PBS, 0.1% (v/v) Triton-X 100 for 20 minutes. Slides were incubated with primary and secondary antibodies as outlined above before image acquisition.

Xenograft tumors and tissue microarrays (Biomax) were formalin fixed and paraffin embedded prior to sectioning and staining. Sections were re-hydrated sequentially from xylene – ethanol – water prior to antigen retrieval by boiling in 10 mM sodium citrate and blocked in TNB (Perkin Elmer). Endogenous peroxidise was blocked with 0.3% Hydrogen Peroxide (H_2_O_2_) prior to all immuno-peroxidase staining protocols. Non-specific binding of secondary antibody was blocked using 3% normal serum from the animal of origin of the corresponding secondary antibody. Slides were incubated in primary antibody (1:100) overnight at 4°C. Secondary antibodies were detected using Avidin Biotin Complex (ABC) reagent (Vector labs), followed by the chromogen 3,3'- Diaminobenzidine (DAB) reagent (Vector labs) as per the manufacturer's instructions. Sections were counterstained with heamatoxylin and imaged under a light microscope (Nikon) or the ScanScope digital slide scanner (Aperio). Immunohistochemical staining was quantified by H-score. Staining intensity was grouped into four categories and a numerical multiplier assigned (bracketed); no stain (0) low intensity (+1), moderate intensity (+2) and high intensity (+3). The percentage of cells, within each staining intensity, was multiplied by the multiplier to give a total H-score for comparison. Scoring was completed on multiple representative fields of view from each sample (n=3). For total scoring of ^p^Ser48NPM slides were scanned with the ScanScope digital slide scanner and total signal intensity and total area of positive staining from the DAB stain quantified by the scanscope software and grouped into scores of no stain, low intensity, moderate intensity and high intensity and scored as above. Further characterization of Pancreatic Ductal Adenocarcinoma was performed under guidance of a pathologist and specific cytoplasmic/nuclear staining was scored by H-Scare as outlined above.

### Molecular Biology and Retrovirus Production

The following plasmids were purchased from Addgene; pBABE puro-myr-FLAGAKT1 (Addgene plasmid 15294), [104], pBABE PuroL myr-HA-AKT2 (Addgene plasmid 9018), pBABE-puro-K-Ras V12 (Addgene plasmid 9052) and pcDNA3 MDM2 S166D S186D (Addgene plasmid 16236). The image clone (IMAGE 6411700, accession number BC054755) encoding mouse Npm was purchased from Source Bioscience. Human NPM was PCR amplified according to standard protocols using the primers sense- aatgaattcatggaagattcgatggacatggacatgagc and antisense- aatctcgagaagagacttcctccactgccagagatcttg and cloned into the C-terminal FLAG tagging vector PCMV 4 (Aligent), between the EcorI and XhoI restriction enzyme sites. Human NPM was PCR amplified using the primers NPM_pbabe_FWD aataatggatccatggaagattcgatggacatgg and NPM_pbabe_REV aataatgaattcttaaagagacttcctccactgcc and cloned into the retroviral vector pBABE Puro between the BamH and EcoI restriction sites. Primers used for mutation of Ser48 to Ala; Hu_NPM_S48A_sense gttatctttaagaacggtcgctttaggggctggtgcaaag & Hu_NPM_S48A_antisense ctttgcaccagcccctaaagcgaccgttcttaaagataac. Primers used for the mutation of Ser48 to Glu Hu_NPM_S48E_sense ccagttatctttaagaacggtcgagttaggggctggtgcaaaggatg and Hu_NPM_S48E_antisense catcctttgcaccagcccctaactcgaccgttcttaaagataactgg. Primers used for mutation of siRNA (ACAAGAAUCCUUCAAGAAA) recognition site sense-catcaacaccaagatcaaaaggacaagagagctttaagaaacaggaaaaaactcctaaaacac & antisense gtgttttaggagttttttcctgtttcttaaagctctcttgtccttttgatcttggtgttgatg. Mouse Npm was amplified by PCR used the primers Mus_Npm_Fwd aataatggatccatggaagactcgatggatatgg & Mus_Npm_Rev aataatgaattcttaaagagatttcctccactgcc and cloned into the pBABE Puro vector between the BamHI and EcorI restriction sites. Primers used for mutagenesis of Ser48 to Ala MusNpm_S48A_sense cagttgtcattaagaacggtcgcgttaggagcaggggcaaaagat & MusNpm_S48A_antisense atcttttgcccctgctcctaacgcgaccgttcttaatgacaactg. Primers used for the mutagenesis of Ser48 to Glu MusNpm_S48E_sense ccagttgtcattaagaacggtcgagttaggagcaggggcaaaagatg and MusNpm_S48E_antisense catcttttgcccctgctcctaactcgaccgttcttaatgacaactgg. Site directed mutagenesis was achieved using the Quikchange II kit (Agilent) according to the manufacturer's instructions.

For retroviral production and infections 6 × 10^6^ 293T cells were seeded into a 10 cm dish 16 Hrs before transfection. 293T cells were transfected with 10 μg pBABE plasmid and 10 μg pCL-Eco packing vector using calcium phosphate according to standard protocols. 1 × 10^5^ *NPM −/− P53−/−* MEFs were seeded on a 10 cm dish 24 hrs before the first infection. Virus containing supernatants were filtered (0.4 μm) and mixed 1:2 with fresh media and polybrene (8 μg/ml final concentration). *NPM −/− P53−/−* MEFs were infected a total of 3 times and 24 hrs after the final infection selected in complete media supplemented with 3 μg/ml puromycin (Sigma). Experiments were performed at least 3 days after selection.

### Quantitative real-time PCR

PSN1 and T24 cell monolayers were treated with MK-2206 or DMSO control as outlined in the figure legend before harvesting. Samples were prepared for quantitative RT-PCR using Power SYBR® Green Cells-to-CT™ Kit (Life Technologies), according to the manufactures protocol. The Real-Time PCR Cycling Conditions were as follows: Holding Stage, 95°C for 10min (×1), Cycling Stage: Step 1- 95°C for 15 sec and Step 2- 60°C for 1 min (× 50), Melt Curve Stage (continuous): Step 1- 95°C for 15 sec, Step 2- 60°C for 1 min, Step 3- 95°C for 30 sec and Step 4- 60°C for 15sec. 18S was used as an internal control to normalize all data. The following primers were used: p53 FW: ACGCTTCCCTGGATTGGCAGC R: GAGGGGGCTCGACGCTAGGA, p14^ARF^ FW: CTACTGAGGAGCCAGCGTCTA R: CTGCCCATCATCATGACCT and 18S FW: AGTCCCTGCCCTTTGTACACA R: GATCCGAGGGCCTCACTAAAC. The experiments were carried out in triplicate for each data point.

### Clonogenic survival curves

In all clonogenic survival experiments, (200-400) cells were plated from single cell suspensions and allowed to adhere to culture dishes prior to irradiation and / or inhibitor exposure. Inhibitor treatment was initiated 1 hour prior to irradiation and maintained for 24 hours. After the treatment interval, the medium was replaced with drug-free medium. Control cultures underwent medium replacement at the same time to control for this manipulation. Cells were irradiated with a Mark 1 cesium irradiator (J.L. Shepherd) at a dose rate of 1.7 Gy/min. Colonies were stained with crystal violet solution and counted 10 to 30 days after irradiation. The surviving fraction was derived using the formula:

(# Colonies / # of cells plated) _irradiated_ / (# Colonies / # of cells plated) _unirradiated_.

Each point on the survival curve represents the mean surviving fraction from at least three dishes. Clonogenic survival curves are representative of independent replicate experiments.

### 3D colony growth assay

3D colony assay of the KPC mouse derived cells was adapted from a previously described protocol for 3D culture of mouse pancreatic cells [105]. Cells were resuspended at a density of 2.5 × 10^3^ cells/0.5 mL in methylcellulose-based colony culture medium. In short, 1 mL of the culture mixture contained DMEM, 1% (wt/vol) methylcellulose (Sigma), 5% (vol/vol) Matrigel (BD Bioscience), 50% (vol/vol) conditioned media from KPC mouse cells in culture, 5% (vol/vol) FCS, 10 mmol/L nicotinamide (Sigma), 10 ng/mL human recombinant activin-βB (R & D Systems), 0.1 nmol/L exendin-4 (Sigma), and 1 ng/mL vascular endothelial growth factor–A. The cells were treated with MK-2206 (1μM, 24hrs) prior to irradiation with a Mark 1 cesium irradiator (J.L. Shepherd) at a dose rate of 1.7 Gy/min for a total of 6 Gy. Colonies were counted after 15 days using a Nikon Eclipse TE2000-E microscope.

### xCELLigence growth assay

KPC mice derived cells were plated at a density of 15000 cells/ml in an E-Plate 16 (ACEA Biosciences,Roche) according to manufacturer's instructions. The growth characteristics were measured using an xCELLigence RTCA DP (ACEA Biosciences,Roche) analyser which recorded the growth in terms of cell index, which is a dimensionless parameter derived as a relative change in measured electrical impedance to represent cell status. Cell Index *_i_*= (R_tn_-R_t0_)/F*_i_* where *i*= 1,2,3 F_1_=15Ω, F_2_=12Ω, F_3_=10Ω and n=0,1,2,…N(time points).

### Resazurin Assay

5 × 10^3^ cells per well was plated in multiples of 6 wells per condition. Fluorescence of Resofurin produced by conversion of Resazurin to Resofurin by viable cells after 48 hours following treatment with 4Gy radiation and / or Doxorubicin (1μM) was read on a plate reader.

### Senescence Experiments

100 ×10^3^ T24 or DLD1 cells were seeded in a 10 cm dish and plated in 10% (v/v) FCS containing medium. Cells were swapped into 0.1% (v/v) FCS containing media and following 24 hrs were treated with 1 μM PI-103 or 5 μM MK-2206 for a further 24 hrs. In some experiments cells were irradiated (4 Gy) after drug treatment. Following drug treatment cells were swapped into fresh 0.1% (v/v) FCS containing media and cultured at 37°C in water saturated 5% CO_2_/95% air. 5 days later, cells were fixed and stained for β-galactosidase activity. Cells were washed twice with PBS and fixed in 2% (v/v) formaldehyde, 0.2% (v/v) glutaraldehyde in PBS (15 min, at room temp). Following fixation, cells were washed with PBS and stained with β-Galactosidase stain solution (1 mg/ml X-gal (5-bromo-4-chloro-indolyl-β-D-galactopyranoside) dissolved in dimethyl-formamide), 40 mM citric acid/sodium phosphate buffer (pH6.0), 5 mM potassium ferricyanide (K_3_Fe(CN)_6_), 5 mM potassium ferrocyanide (K_4_Fe(CN)_6_.3H_2_O), 150 mM NaCl, 2 mM MgCl_2_) for 12-16 Hrs at 37ºC. Cells were viewed under a light microscope and those with β-galactosidase positivity and large cell morphology indicative of senescence were counted.

### *In-vivo* xenografts

All animal procedures were performed in accordance with current UK legislation under an approved project license. Female athymic nude mice (BALB/c nude) (Harlan) were divided into groups receiving injections subcutaneously (s.c) into the flank with 1 × 10^6^ PSN-1 HRE luc human PCC cells with 4 × 10^6^ LTC-14 (stellate cells). Animals were assigned randomly into different groups, to receive carrier (β- cyclo-dextrin (1.5mg/ml)), 60 mg/kg, and 120 mg/kg of MK-2206 s/c on three alternate days and 320 mg/kg of MK-2206 s/c once, in the first experiment. Treatments were initiated when tumors reached 100 mm^3^. Animals were assigned randomly into different groups in the second experiment to receive either carrier or 60 mg/kg of MK-2206 s/c on two alternate days followed by irradiation, a 6 Gy single dose under anaesthesia on day 4. Tumor growth was measured regularly by calipers. MK-2206 was made up in β- cyclo-dextrin (1.5mg/ml) for in vivo experiments at the time of randomization of animals and any made up drug discarded after last dose of drug was injected. For SQ20B xenografts PI-103 treatment, female severe combined immunodeficient (SCID) mice (Charles River) were inoculated with 10^6^ SQ20B cells on the hind leg s.c. Treatments were initiated when tumors reached 100 mm^3^. In all experiments, animals were treated with carrier (50% DMSO, 50% PBS) or PI-103 (5 mg/kg) by daily i.p. injections. Inhibitors were given daily for up to 2 wk.

## SUPPLEMENTARY MATERIAL FIGURES NAD TABLES


