# Supplementary material for: AKT regulates NPM dependent ARF localization and p53mut stability in tumors
Source: Oncotarget. 2014 Jul 8;5(15):6142–67. doi: 10.18632/oncotarget.2178 (PMC4171619; doi:10.18632/oncotarget.2178)
Supplement: Supplementary file 1 [file oncotarget-05-6142-s001.pdf]

AKT regulates NPM dependent ARF localization and p53mut stability in tumors

SUPPLEMENTARY MATERIAL

Experimental procedures

Cell culture and transfection: HT-1080-SG1 and SG2 tumor cell lines were kindly provided by Eric Stanbridge (Stanford, CA), AKT knockout MEF's were kindly provided by Dr. Birnbaum (University of Pennsylvania PA). *NPM*$^{-/-}$, *p53*$^{-/-}$ and *p53* $^{-/-}$ MEFs were kindly provided by Pier Paolo Pandolfi (Harvard, Boston MA). PSN1 cells were kindly provided by Thomas Brunner (University of Oxford) and subsequently genotyped at the DDC laboratories, London, to confirm cell identity. The mutation data reflects the information on the COSMIC Cell Line Project database http://cancer.sanger.ac.uk/cancergenome/projects/cell_lines/. All other cell lines were obtained from the American Type Culture Collection (ATCC). Cells were cultured in DMEM containing 4.5 g/l glucose (Invitrogen) supplemented with 10 % fetal bovine serum (Invitrogen), penicillin (100 units/ml), and streptomycin (100 mg/ml). All cultures were maintained at 37°C in water saturated 5 % CO_2 with the exception of MRC5 cells which were cultured at 37°C in 3 % O_2/5 % CO_2. Cells were plated to 80 % confluence prior to siRNA transfection. Smartpool siRNA (Dharmacon) against NPM, p14^{ARF} and AKT were used where indicated. Individual siRNA against NPM (ACAAGAAUCCUUCAAGAAA) was used in conjunction with re-expression of NPM constructs. sip73 sequences (GCAAGCAGCCCAUCAAGGA and GAGACGAGGACACGUACUA), sip53 (GACUCCAGUGGUAAUCUAC), siULF (GGUAGUGACUCCACCCAUUUU). Cells were transfected with plasmid or siRNA (50 nM) construct using Lipofectamine 2000 (Invitrogen) or Dharmafect (Dharmacon) for 48 hours prior to drug treatment. Mutant p53 plasmids (R248W & R175H) cloned into pcDNA3 vector backbone were a kind gift from Xin Lu (Ludwig Institute for Cancer Research, University of Oxford).

KPC mice derived cell lines: *Pdx1-cre*, *KrasG12D*, *Trp53flox*, *Trp53R172H* and *ARF-/-* mice have been described previously [1-5] . Mice were kept in conventional animal facilities and experiments were carried out in compliance with UK Home Office guidelines. Genotyping was performed by Transnetyx (Cordova, TN, USA). Animals were monitored until showing symptoms of late stage pancreatic cancer and then sacrificed as per institutional guidelines. Tumor and metastatic burden was assessed by gross pathology and histology.

Tumor tissue for preparation of PDAC cell lines was harvested in DMEM. Tumors were disaggregated by fine mincing with scalpels, and plated in growth media (Dulbecco's modified Eagle medium containing 10% fetal bovine serum and 2mmol/L L-glutamine). Cells were allowed to adhere, washed, grown to confluence and then passaged as normal.

Chemicals and antibodies: All chemicals were purchased from Sigma Aldrich unless stated otherwise. PI-103 was purchased from Merck. CCI-779 was purchased from Sigma. MK-2206 was purchased from ChemieTek. All inhibitors were dissolved as concentrated stock solutions in DMSO (1 mM) and stored at -80°C. Antibodies, anti-AKT1 (#9272), anti-AKT2 (#2964), anti-AKT1 (#2938) anti-phospho-Ser 473-AKT (#9721), anti-p44/42 MAPK (Erk1/2) (#137F5), anti-phospho-p44/42 MAPK (Erk1/2) (Thr202/Tyr204) (#9106) and anti-phospho-S6 (Ser 235/236) (#91B2) were purchased from Cell Signaling Technologies. Anti-GAPDH (2251-1) and anti-p73 (1636-1) were purchased from Epitomics. Anti-HA tag (05-904) and anti-Myc tag (05-724) were purchased from Millipore. Anti-p14^{ARF} (Ab11048 and Ab49166), anti-nucleophosmin (ab10530), anti-ULF (Ab80645), anti-Fibrillarin (Ab5821), anti-Lamin B1 (Ab16048) and anti-p53 (D01 clone) (Ab80645), were purchased from Abcam. Anti-p19^{ARF} (NB-200-174) and anti-p14^{ARF} (NB-200-111) were purchased from Novus Biologicals. Anti-FLAG (M2) was purchased from Agilent Technologies. Anti-actin (A4700) was purchased from Sigma. Anti-p21 (sc6246), anti-MDM2 (SMP14 (Sc-965)), anti-HSP-90 (Sc-69703) and anti-Bax (2D2) were purchased from Santa Cruz Biotechnology. The anti phospho-Ser48-NPM was raised against a synthetic peptide spanning residues 45-56 of NPM (RTVSLGAGAKDE) incorporating phospho Ser (underlined) at

position 48. Peptide synthesis and immunizations were carried out by Eurogentec. The anti phospho-Ser48-NPM used in this study was affinity purified against the phosphopeptide. Secondary antibodies anti-mouse and anti-rabbit HRP conjugates were purchased from Pierce and the Jackson Laboratory. Fluorescent tagged antibodies for use on the Licor Odyssey western blot imaging system were purchased from Licor and Invitrogen. Fluorescent conjugated secondary antibodies for immunofluorescence were purchased from Invitrogen.

Protein biochemistry

Preparation of cell lysates and western blot: Unless specified otherwise, whole cell lysates were prepared by lysing cells with 1% NP-40 lysis buffer (50 mM Tris-HCl, 150 mM NaCl, 2 mM EGTA, 5 mM $MgCl_2$, 1 % (v/v) NP-40, 10 mM sodium β-glycerophosphate, 50 mM NaF, 1 mM Na_3VO_4, 5 mM sodium pyrophosphate and 'Complete' proteinase inhibitor cocktail EDTA free (1 tablet/10 ml lysis buffer (Roche)). Lysates were rotated end over end for 30 min at 4ºC and centrifuged (20,817 x g, 10 min) before the addition of NuPage sample buffer or SDS-PAGE sample buffer (1x concentration, 62.5 mM Tris-HCl pH 6.8, 25 % (v/v) glycerol, 2 % (w/v) SDS, 0.01 % (w/v) bromophenol blue). For immunoprecipitation, lysates were pre-cleared (4 ºC, 1 Hr) with protein-G coupled to magnetic beads (Millipore), prior to incubation with antibody conjugated to protein-G magnetic beads. Lysates and antibody coupled beads were rotated end over end at 4°C for at least 3 Hrs. Immunoprecipitates were washed (4 x 1ml) with lysis buffer minus the protease and phosphatase inhibitors. Immunoprecipitated proteins were boiled in SDS-PAGE sample buffer for western blot analysis.

Nuclear lysates were prepared as described previously [6] with additional modifications. Briefly, 3-5 x 10^6 cells were trypsinised and harvested by centrifugation (500 x g, 5 min) , washed twice in TBS and re-suspended in 1-2 ml ice cold buffer A (10 mM HEPES pH 7.9, 10 mM KCl, 0.1 mM EDTA, 0.1 mM EGTA, 1 mM DTT, 0.5 mM PMSF) by gently pipetting in a 1 ml tip. The cells were left on ice for 15 min to swell, after which 75 µl of 10 % NP-40 was added. The tube was vigorously vortexed for 10 sec and

centrifuged at 500 x g for 2 min. The supernatant, which constitutes the cytoplasmic fraction, was removed. The nuclear pellet was re-suspended in 150 µl ice-cold lysis buffer (150 mM NaCl, 20 mM Hepes pH 7.5, 0.5 mM EDTA, 0.5% (v/v) NP-40, 10 mM sodium β-glycerophosphate, 50 mM NaF, 1 mM Na_3VO_4, 5 mM sodium pyrophosphate and 'Complete' proteinase inhibitor cocktail EDTA free (1 tablet/10 ml lysis buffer) and the sample sonicated. The nuclear extract was centrifuged (20,817 x g, 15 min, 4^0C) and the supernatant containing the nuclear extract was used as an in-put for immunoprecipitation or added to SDS-PAGE sample buffer as described above.

For western blot, samples were boiled (100 ºC, 5 min) and proteins resolved on NuPage Bis-Tris gels (Invitrogen). Resolved proteins were transferred by western blot to PVDF (Millipore) or Nitrocellulose (Biorad) membrane and blocked in either 5% (w/v) non-fat milk or 5 % (w/v) BSA dissolved in PBS, 0.1 % (v/v) Tween-20 prior to antibody addition. Those membranes probed for phosphorylated proteins were blocked in 5 % (w/v) BSA, TBS (50 mM Tris-HCl, pH 7.5, 150 mM NaCl), 0.1 % (v/v) Tween-20. Primary antibody detection was achieved with HRP conjugated secondary antibodies (Pierce) and exposure to X-Ray film (Kodak). For blots which were quantified, samples were run by western blot and transferred onto Nitrocellulose membrane (Biorad). Membranes were blocked in Licor blocking buffer and incubated with primary antibody overnight followed by incubation with fluorescently conjugated secondary antibodies. Membranes were scanned on the Licor Odyssey infrared scanner and signal intensity determined using the Odyssey software (V3.0). Signals were normalized to GAPDH as a loading control. All quantification was done on the same nitrocellulose membrane without stripping.

***In-Vitro* Kinase assay:** Endogenous AKT was immunoprecipitated from T24 cells, glycine eluted and combined with immunoprecipitated HA-NPM, HA-NPM-S48A or anti HA-IP from non-transfected cells as indicated in 1 x kinase buffer (Cell Signaling). The reaction was incubated with cold ATP (20 µM) and radio-labeled gamma ^{32}P ATP (2 µM) at 37°C. The reaction was heat inactivated in the presence of denaturing SDS-PAGE sample buffer, separated by SDS-PAGE, western blotted and exposed to a phosphor screen. The membrane was additionally probed by standard western blot.

Ubiquinitation assays: H1299 cells (1 x 10^6) were transfected with pcDNA3 expressing either HA-tagged Ubiquitin or Myc-tagged Ubiquitin (10 µg) alone or in combination with pcDNA3 expressing $p53^{Wt}$ (10 µg) or the p53 mutants R175H or R248W (10 µg) using Lipofectamine 2000 (Invitrogen). 24 hrs following transfection cells were treated with 10 µM MG-132, 5 µM MK-2206 or 5 µM Nutlin3A as indicated in the figure legends. Cells were washed twice with PBS and lysed by scrapping in ice cold RIPA buffer (50 mM Tris pH 8.0, 150 mM NaCl, 1 % (v/v) Triton X 100, 0.5 % (w/v) sodium deoxycholate, 0.1 % (w/v) SDS, 1 mM EDTA, 1 mM EGTA, 50 mM NaF, 1 mM Na_3V0_4, 10 mM sodium β-glycerophosphate, 5 mM sodium pyrophosphate and 'Complete' proteinase inhibitor cocktail EDTA Free (1 tablet / 10ml lysis buffer) supplemented with 1 mM N-Ethylmaleimide. Lysates were rotated end over end (4ºC, 30 min), sonicated, centrifuged (20,817x g, 15 min, 4ºC) and pre-cleared with protein G coupled to magnetic beads (Millipore). Pre-cleared lysates were incubated with the appropriate antibody conjugated to protein G for 3 Hrs. Protein G beads were washed with 50 mM Tris pH 8.0, 150 mM NaCl, 1 % (v/v) Triton-X-100, 0.5 % (w/v) sodium deoxycholate, 0.1 % (w/v) SDS before boiling in SDS-PAGE sample buffer. Samples were resolved on 10 % NuPage Bis-Tris gels or 4-8 % Nupage Tris-Acetate gels (Invitrogen).

Semi-native Gel Electrophoresis: Cells were washed twice with ice cold PBS and lysed by scrapping in ice cold lysis buffer (50 mM Tris pH 8.0, 150 mM NaCl, 1 % (v/v) Triton X 100, 1 mM EDTA, 1 mM EGTA, 50 mM NaF, 1 mM Na_3V0_4, 10 mM sodium β-glycerophosphate, 5 mM sodium pyrophosphate and 'Complete' proteinase inhibitor cocktail EDTA Free (1 tablet / 10ml lysis buffer). Lysates were rotated end over end (4ºC, 30 min), centrifuged (21000 x g. 15 min), diluted in the appropriate volume of NuPage sample buffer (samples were not boiled) and immediately loaded onto 10 % Bis-Tris Nupage gels or Native Nupage gels. Gels were run at a constant voltage (100 V) at 4ºC before transfer to Nitrocellulose membrane.

Purification of $p14^{ARF}$ from HeLa Nuclear extracts: Fractionation of HeLa cells was performed as previously described [7]. Briefly, 20 grams of HeLa cell pellets (Cilbiotech, Mons, Belgium) were

resuspended in 40 ml lysis buffer (20 mM Tris–HCl pH 7.4, 2.5 mM $MgCl_2$, 0.5 % (v/v) Nonidet P-40, 1 mM PMSF, 1 mM DTT and 1 mg/ml each of aprotinin, pepstatin, chymostatin and leupeptin) and incubated on ice for 10 min prior to centrifugation (1,300 × g, 4 min at 4°C). The supernatant containing cytoplasmic proteins was discarded and the remaining cell pellet was further resuspended in 40 ml buffer containing 100 mM phosphate, pH 8.0, 0.5 M KCl, 5 mM $MgCl_2$, 1 mM EDTA, 0.75 % (v/v) Triton X-100, 10% (v/v) glycerol, 1 mM PMSF, 1 mM DTT and 1 mg/ml each of aprotinin, pepstatin, chymostatin and leupeptin and the supernatant containing nuclear proteins was collected. The obtained extract was dialyzed against Buffer A (50 mM Tris-HCl, pH 8.0, 1 mM EDTA, 5 % (v/v) glycerol, 1 mM DTT and 0.1 mM PMSF) containing 50 mM KCl. The extract was applied to a 20 ml HiLoad Q Sepharose column (GE Healthcare). The column was washed in Buffer A containing 300 mM KCl and proteins bound to the column were eluted using a linear gradient of 300-700 mM KCl. Fractions containing $p14^{ARF}$ (fractions B8-B2) were pooled, concentrated using Amicon Ultra Ultracel-3K filter units (Millipore) and separated on a Superdex 200 HR 10/30 column (GE Healthcare) in Buffer A containing 150 mM KCl. Fractions containing $p14^{ARF}$ were eluted as two separate pools of different molecular weight (pool I is represented by fractions B6-C1, pool II consists of fractions C4-C10) were pooled, the fractions in each pool were combined and further loaded separately onto a 1 ml MonoQ column (GE Healthcare) in Buffer A containing 150 mM KCl. The column was washed and bound proteins were eluted as described above for the HiLoad Q Sepharose column purification step. At each purification step, aliquots of the obtained fractions were analyzed by western blot for the presence of $p14^{ARF}$ using a specific antibody (Bethyl Laboratories, A300-340A). Fractions identified as containing $p14^{ARF}$ were pooled for the next chromatography step.

35S Met/Cys Pulse Chase: T24 cells (1 x 10^6/10 cm dish) were plated 24 hrs before addition of MK-2206 (5 µM) or DMSO control and incubated for 18 Hrs. MK-2206 or DMSO was maintained in culture media throughout the experiment Cells were washed twice with Met/Cys free media (Invitrogen) and cultured for 1 hr at 37ºC with Met/Cys free media. Following depletion of intracellular Met/Cys stores, cells were

incubated with Met/Cys free media supplemented with 200 µCi ^{35}S Met/Cys (EasyTag™ EXPRESS35S Protein Labeling Mix 35S, PerkinElmer). Cells were metabolically labeled for 1 Hr before being washed twice with complete media containing unlabeled Met/Cys. Cells were chased for 30 min- 4 Hrs in complete media at 37ºC before being washed twice with PBS and lysed by scrapping in 1 % (v/v) NP-40 lysis buffer. p53 was immunoprecipitated as outlined above. Samples were run on a 10 % NuPage Bis Tris gel. Gels were dried before exposure to a phosphor screen. Additionally samples were probed by western blot.

Immunofluorescence and Immunohistochemistry: For analysis of cells using the In Cell Analyzer 1000, automated epifluorescence microscope (GE Healthcare), cells were plated into 96-well plates at a density of 10,000 cells/well and incubated overnight at 37°C with 5% CO_2 in a humidified incubator. Cells were exposed to inhibitor 24 hours prior to fixation. Cells were fixed with 4 % paraformaldehyde and permeabilized with 1 % (v/v) TritonX-100 and blocked with a 1 (w/v) % solution of BSA in PBS. Cells were incubated with primary antibody as indicated (1:1000 dilution), overnight at 4°C. Primary antibody was detected using Alexafluor conjugate secondary antibodies (Invitrogen) at 1:1000 dilution. Cells were counterstained with DAPI (1 µg/ml). Foci were detected using an In Cell Analyzer 1000 automated epifluorescence microscope (GE Healthcare). For all other immunofluorescence based experiments cells were grown on coverslips, fixed with 4 % (v/v) PFA and permeabilized with 0.1 % (v/v) Triton X-100. Coverslips were blocked with 3 % (w/v) BSA dissolved in PBS and incubated with primary antibody (1/100) prepared in blocking buffer, overnight at 4ºC. Coverslips were washed with PBS and incubated with the appropriate fluorescently conjugated secondary antibody (1/500) for 1 Hr at room temperature. Coverslips were washed (3 X PBS) and images captured using a Nikon 90i epifluorescent microscope or LSM 710 (Zeiss) confocal microscope.

For frozen tissue sections, slides were fixed in acetone for 10 minutes at room temperature. Slides were dried, washed in PBS and non-specific binding blocked with 3% (v/v) normal bovine serum (NBS), PBS,

0.1 % (v/v) Triton-X 100 for 20 minutes. Slides were incubated with primary and secondary antibodies as outlined above before image acquisition.

Xenograft tumors and tissue microarrays (Biomax) were formalin fixed and paraffin embedded prior to sectioning and staining. Sections were re-hydrated sequentially from xylene – ethanol – water prior to antigen retrieval by boiling in 10 mM sodium citrate and blocked in TNB (Perkin Elmer). Endogenous peroxidise was blocked with 0.3 % Hydrogen Peroxide (H_2O_2) prior to all immuno-peroxidase staining protocols. Non-specific binding of secondary antibody was blocked using 3 % normal serum from the animal of origin of the corresponding secondary antibody. Slides were incubated in primary antibody (1:100) overnight at 4°C. Secondary antibodies were detected using Avidin Biotin Complex (ABC) reagent (Vector labs), followed by the chromogen 3,3'- Diaminobenzidine (DAB) reagent (Vector labs) as per the manufacturer's instructions. Sections were counterstained with heamatoxylin and imaged under a light microscope (Nikon) or the ScanScope digital slide scanner (Aperio). Immunohistochemical staining was quantified by H-score. Staining intensity was grouped into four categories and a numerical multiplier assigned (bracketed); no stain (0) low intensity (+1), moderate intensity (+2) and high intensity (+3). The percentage of cells, within each staining intensity, was multiplied by the multiplier to give a total H-score for comparison. Scoring was completed on multiple representative fields of view from each sample (n=3). For total scoring of ^{P}Ser48NPM slides were scanned with the ScanScope digital slide scanner and total signal intensity and total area of positive staining from the DAB stain quantified by the scanscope software and grouped into scores of no stain, low intensity, moderate intensity and high intensity and scored as above. Further characterization of Pancreatic Ductal Adenocarcinoma was performed under guidance of a pathologist and specific cytoplasmic/nuclear staining was scored by H-Scare as outlined above.

Molecular Biology and Retrovirus Production: The following plasmids were purchased from Addgene; pBABE puro-myr-FLAGAKT1 (Addgene plasmid 15294), [8], pBABE PuroL myr-HA-AKT2 (Addgene plasmid 9018), pBABE-puro-K-Ras V12 (Addgene plasmid 9052) and pcDNA3 MDM2 S166D S186D

(Addgene plasmid 16236). The image clone (IMAGE 6411700, accession number BC054755) encoding mouse Npm was purchased from Source Bioscience. Human NPM was PCR amplified according to standard protocols using the primers sense- aatgaattcatggaagattcgatggacatggacatgagc and antisense-aatctcgagaagagacttcctccactgccagagatcttg and cloned into the C-terminal FLAG tagging vector PCMV 4 (Aligent), between the EcorI and XhoI restriction enzyme sites. Human NPM was PCR amplified using the primers NPM_pbabe_FWD aataatggatccatggaagattcgatggacatgg and NPM_pbabe_REV aataatgaattcttaaagagacttcctccactgcc and cloned into the retroviral vector pBABE Puro between the BamH and EcoI restriction sites. Primers used for mutation of Ser48 to Ala; Hu_NPM_S48A_sense gttatctttaagaacggtcgctttaggggctggtgcaaag & Hu_NPM_S48A_antisense ctttgcaccagcccctaaagcgaccgttcttaaagataac. Primers used for the mutation of Ser48 to Glu Hu_NPM_S48E_sense ccagttatctttaagaacggtcgagttaggggctggtgcaaaggatg and Hu_NPM_S48E_antisense catcctttgcaccagcccctaactcgaccgttcttaaagataactgg. Primers used for mutation of siRNA (ACAAGAAUCCUUCAAGAAA) recognition site sense-catcaacaccaagatcaaaaggacaagagagctttaagaaacaggaaaaaactcctaaaacac & antisense gtgttttaggagtttttcctgtttcttaaagctctcttgtccttttgatcttggtgttgatg. Mouse Npm was amplified by PCR used the primers Mus_Npm_Fwd aataatggatccatggaagactcgatggatatgg & Mus_Npm_Rev aataatgaattcttaaagagatttcctccactgcc and cloned into the pBABE Puro vector between the BamHI and EcorI restriction sites. Primers used for mutagenesis of Ser48 to Ala MusNpm_S48A_sense cagttgtcattaagaacggtcgcgttaggagcaggggcaaaagat & MusNpm_S48A_antisense atcttttgcccctgctcctaacgcgaccgttcttaatgacaactg. Primers used for the mutagenesis of Ser48 to Glu MusNpm_S48E_sense ccagttgtcattaagaacggtcgagttaggagcaggggcaaaagatg and MusNpm_S48E_antisense catcttttgcccctgctcctaactcgaccgttcttaatgacaactgg. Site directed mutagenesis was achieved using the Quikchange II kit (Agilent) according to the manufacturer's instructions.

For retroviral production and infections 6 x 10^6 293T cells were seeded into a 10 cm dish 16 Hrs before transfection. 293T cells were transfected with 10 µg pBABE plasmid and 10 µg pCL-Eco packing vector

using calcium phosphate according to standard protocols. 1 x 10^5 *NPM -/- P53-/-* MEFs were seeded on a 10 cm dish 24 hrs before the first infection. Virus containing supernatants were filtered (0.4 µm) and mixed 1:2 with fresh media and polybrene (8 µg/ml final concentration). *NPM -/- P53-/-* MEFs were infected a total of 3 times and 24 hrs after the final infection selected in complete media supplemented with 3 µg/ml puromycin (Sigma). Experiments were performed at least 3 days after selection.

Quantitative real-time PCR: PSN1 and T24 cell monolayers were treated with MK-2206 or DMSO control as outlined in the figure legend before harvesting. Samples were prepared for quantitative RT-PCR using Power SYBR® Green Cells-to-CT™ Kit (Life Technologies), according to the manufactures protocol. The Real-Time PCR Cycling Conditions were as follows: Holding Stage, 95°C for 10min (x1), Cycling Stage: Step 1- 95°C for 15 sec and Step 2- 60°C for 1 min (x 50), Melt Curve Stage (continuous): Step 1- 95°C for 15 sec, Step 2- 60°C for 1 min, Step 3- 95°C for 30 sec and Step 4- 60°C for 15sec. 18S was used as an internal control to normalize all data. The following primers were used: p53 FW: ACGCTTCCCTGGATTGGCAGC R: GAGGGGGCTCGACGCTAGGA, p14^{ARF} FW: CTACTGAGGAGCCAGCGTCTA R: CTGCCCATCATCATGACCT and 18S FW: AGTCCCTGCCCTTTGTACACA R: GATCCGAGGGCCTCACTAAAC. The experiments were carried out in triplicate for each data point.

Clonogenic survival curves: In all clonogenic survival experiments, (200-400) cells were plated from single cell suspensions and allowed to adhere to culture dishes prior to irradiation and / or inhibitor exposure. Inhibitor treatment was initiated 1 hour prior to irradiation and maintained for 24 hours. After the treatment interval, the medium was replaced with drug-free medium. Control cultures underwent medium replacement at the same time to control for this manipulation. Cells were irradiated with a Mark 1 cesium irradiator (J.L. Shepherd) at a dose rate of 1.7 Gy/min. Colonies were stained with crystal violet solution and counted 10 to 30 days after irradiation. The surviving fraction was derived using the formula:

(# Colonies / # of cells plated) $_{\text{irradiated}}$ / (# Colonies / # of cells plated) $_{\text{unirradiated}}$.

Each point on the survival curve represents the mean surviving fraction from at least three dishes. Clonogenic survival curves are representative of independent replicate experiments.

3D colony growth assay: 3D colony assay of the KPC mouse derived cells was adapted from a previously described protocol for 3D culture of mouse pancreatic cells [9]. Cells were resuspended at a density of 2.5×10^3 cells/0.5 mL in methylcellulose-based colony culture medium. In short, 1 mL of the culture mixture contained DMEM, 1% (wt/vol) methylcellulose (Sigma), 5% (vol/vol) Matrigel (BD Bioscience), 50% (vol/vol) conditioned media from KPC mouse cells in culture, 5% (vol/vol) FCS, 10 mmol/L nicotinamide (Sigma), 10 ng/mL human recombinant activin-βB (R & D Systems), 0.1 nmol/L exendin-4 (Sigma), and 1 ng/mL vascular endothelial growth factor–A. The cells were treated with MK-2206 (1µM, 24hrs) prior to irradiation with a Mark 1 cesium irradiator (J.L. Shepherd) at a dose rate of 1.7 Gy/min for a total of 6 Gy. Colonies were counted after 15 days using a Nikon Eclipse TE2000-E microscope.

xCELLigence growth assay: KPC mice derived cells were plated at a density of 15000 cells/ml in an E-Plate 16 (ACEA Biosciences,Roche) according to manufacturer's instructions. The growth characteristics were measured using an xCELLigence RTCA DP (ACEA Biosciences,Roche) analyser which recorded the growth in terms of cell index, which is a dimensionless parameter derived as a relative change in measured electrical impedance to represent cell status. Cell Index $_i = (R_{tn}-R_{t0})/F_i$ where i= 1,2,3 F_1=15Ω, F_2=12Ω, F_3=10Ω and n=0,1,2,…N(time points).

Resazurin Assay: 5×10^3 cells per well was plated in multiples of 6 wells per condition. Fluorescence of Resofurin produced by conversion of Resazurin to Resofurin by viable cells after 48 hours following treatment with 4Gy radiation and / or Doxorubicin (1µM) was read on a plate reader.

Senescence Experiments: 100 x10^3 T24 or DLD1 cells were seeded in a 10 cm dish and plated in 10 % (v/v) FCS containing medium. Cells were swapped into 0.1 % (v/v) FCS containing media and following 24 hrs were treated with 1 µM PI-103 or 5 µM MK-2206 for a further 24 hrs. In some experiments cells were irradiated (4 Gy) after drug treatment. Following drug treatment cells were swapped into fresh 0.1 % (v/v) FCS containing media and cultured at 37°C in water saturated 5% CO_2/95% air. 5 days later, cells were fixed and stained for β-galactosidase activity. Cells were washed twice with PBS and fixed in 2 % (v/v) formaldehyde, 0.2 % (v/v) glutaraldehyde in PBS (15 min, at room temp). Following fixation, cells were washed with PBS and stained with β-Galactosidase stain solution (1 mg/ml X-gal (5-bromo-4-chloro-indolyl-β-D-galactopyranoside) dissolved in dimethyl-formamide), 40 mM citric acid/sodium phosphate buffer (pH6.0), 5 mM potassium ferricyanide ($K_3Fe(CN)_6$), 5 mM potassium ferrocyanide ($K_4Fe(CN)_6.3H_2O$), 150 mM NaCl, 2 mM $MgCl_2$) for 12-16 Hrs at 37ºC. Cells were viewed under a light microscope and those with β-galactosidase positivity and large cell morphology indicative of senescence were counted.

***In-vivo* xenografts:** All animal procedures were performed in accordance with current UK legislation under an approved project license. Female athymic nude mice (BALB/c nude) (Harlan) were divided into groups receiving injections subcutaneously (s.c) into the flank with 1 x 10^6 PSN-1 HRE luc human PCC cells with 4 x 10^6 LTC-14 (stellate cells). Animals were assigned randomly into different groups, to receive carrier (β- cyclo-dextrin (1.5mg/ml)), 60 mg/kg, and 120 mg/kg of MK-2206 s/c on three alternate days and 320 mg/kg of MK-2206 s/c once, in the first experiment. Treatments were initiated when tumors reached 100 mm^3. Animals were assigned randomly into different groups in the second experiment to receive either carrier or 60 mg/kg of MK-2206 s/c on two alternate days followed by irradiation, a 6 Gy single dose under anaesthesia on day 4. Tumor growth was measured regularly by calipers. MK-2206 was made up in β- cyclo-dextrin (1.5mg/ml) for in vivo experiments at the time of randomization of animals and any made up drug discarded after last dose of drug was injected. For SQ20B xenografts PI-103 treatment, female severe combined immunodeficient (SCID) mice (Charles River) were inoculated with

10^6 SQ20B cells on the hind leg s.c. Treatments were initiated when tumors reached 100 mm^3. In all experiments, animals were treated with carrier (50% DMSO, 50% PBS) or PI-103 (5 mg/kg) by daily i.p. injections. Inhibitors were given daily for up to 2 wk.

Supplementary References

1. Hingorani SR, Petricoin EF, Maitra A, Rajapakse V, King C, Jacobetz MA, Ross S, Conrads TP, Veenstra TD, Hitt BA, Kawaguchi Y, Johann D, Liotta LA, Crawford HC, Putt ME, Jacks T, et al. Preinvasive and invasive ductal pancreatic cancer and its early detection in the mouse. Cancer cell. 2003; 4(6):437-450.
2. Jackson EL, Willis N, Mercer K, Bronson RT, Crowley D, Montoya R, Jacks T and Tuveson DA. Analysis of lung tumor initiation and progression using conditional expression of oncogenic K-ras. Genes & development. 2001; 15(24):3243-3248.
3. Jonkers J, Meuwissen R, van der Gulden H, Peterse H, van der Valk M and Berns A. Synergistic tumor suppressor activity of BRCA2 and p53 in a conditional mouse model for breast cancer. Nature genetics. 2001; 29(4):418-425.
4. Olive KP, Tuveson DA, Ruhe ZC, Yin B, Willis NA, Bronson RT, Crowley D and Jacks T. Mutant p53 gain of function in two mouse models of Li-Fraumeni syndrome. Cell. 2004; 119(6):847-860.
5. Kamijo T, Zindy F, Roussel MF, Quelle DE, Downing JR, Ashmun RA, Grosveld G and Sherr CJ. Tumor suppression at the mouse INK4a locus mediated by the alternative reading frame product p19ARF. Cell. 1997; 91(5):649-659.
6. Schreiber E, Matthias P, Muller MM and Schaffner W. Rapid detection of octamer binding proteins with 'mini-extracts', prepared from a small number of cells. Nucleic acids research. 1989; 17(15):6419.
7. Woodhouse BC, Dianova, II, Parsons JL and Dianov GL. Poly(ADP-ribose) polymerase-1 modulates DNA repair capacity and prevents formation of DNA double strand breaks. DNA repair. 2008; 7(6):932-940.
8. Boehm JS, Zhao JJ, Yao J, Kim SY, Firestein R, Dunn IF, Sjostrom SK, Garraway LA, Weremowicz S, Richardson AL, Greulich H, Stewart CJ, Mulvey LA, Shen RR, Ambrogio L, Hirozane-Kishikawa T, et al. Integrative genomic approaches identify IKBKE as a breast cancer oncogene. Cell. 2007; 129(6):1065-1079.
9. Jin L, Feng T, Shih HP, Zerda R, Luo A, Hsu J, Mahdavi A, Sander M, Tirrell DA, Riggs AD and Ku HT. Colony-forming cells in the adult mouse pancreas are expandable in Matrigel and form endocrine/acinar colonies in laminin hydrogel. Proceedings of the National Academy of Sciences of the United States of America. 2013; 110(10):3907-3912.
10. Plattner R, Anderson MJ, Sato KY, Fasching CL, Der CJ and Stanbridge EJ. Loss of oncogenic ras expression does not correlate with loss of tumorigenicity in human cells. Proceedings of the National Academy of Sciences of the United States of America. 1996; 93(13):6665-6670.
11. Lee HH, Kim HS, Kang JY, Lee BI, Ha JY, Yoon HJ, Lim SO, Jung G and Suh SW. Crystal structure of human nucleophosmin-core reveals plasticity of the pentamer-pentamer interface. Proteins. 2007; 69(3):672-678.

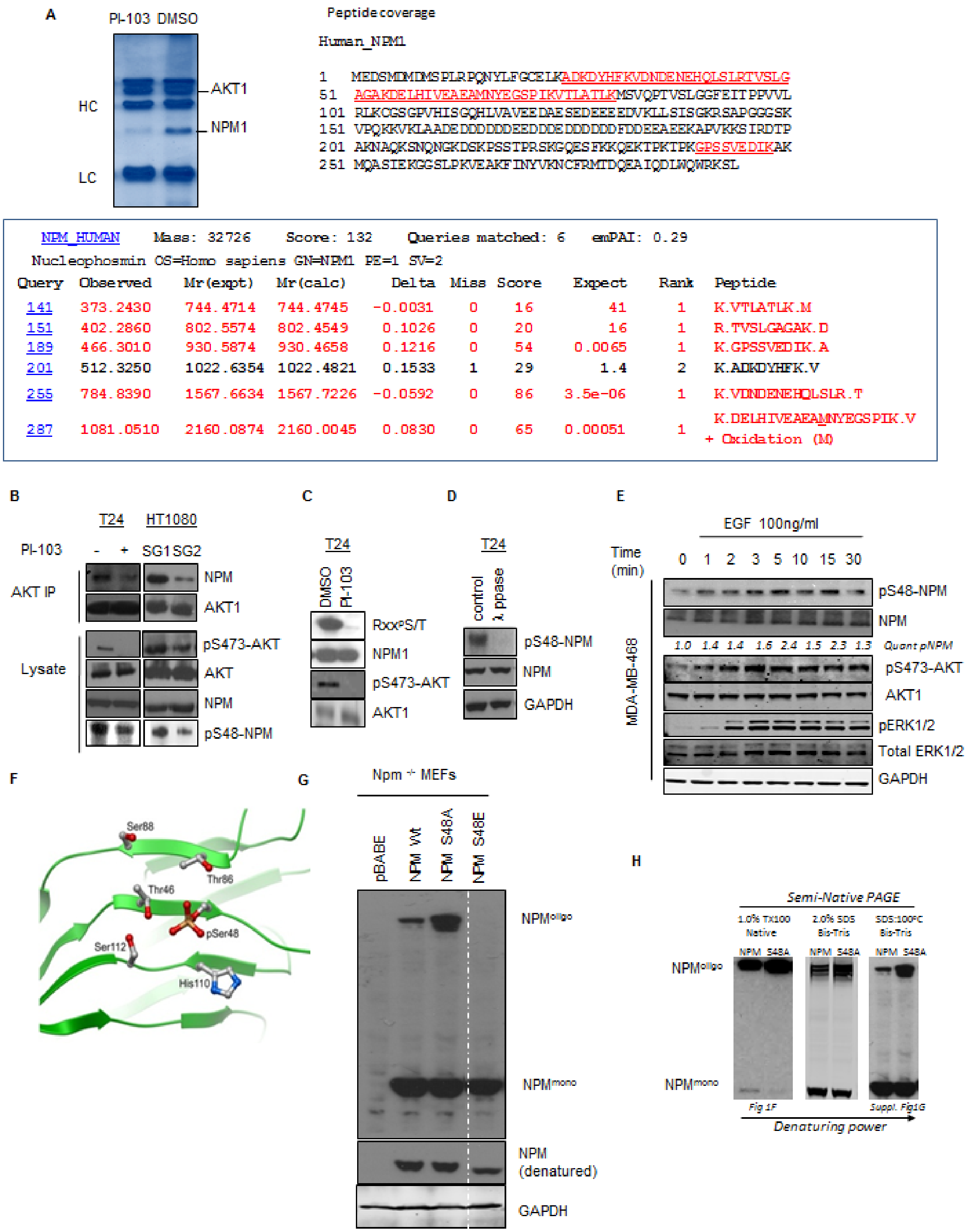

Fig. S1: AKT phosphorylates NPM at S48 which regulates its tertiary structure. (A) Upper left panel shows AKT immunoprecipitates from T24 cells separated by 10% SDS PAGE and stained with colloidal comassie. Gel bands deemed to be associated with AKT were excised and

subjected to tryptic digest followed by separation via HPLC peptide gradient on a pepmap C18 column (Dionex Ultima U300 LC system). Mass Spectrometry was performed on peptides via time of flight in a Bruker Daltonics, Esquire nanoelectrospray ionisation (ESI) High Capacity ion Trap (HCT). Upper right panel shows the peptide coverage of the human NPM1 including Serine 48 (highlighted in red). Lower panel shows the scores for the various peptides including a peptide covering Serine 48 (B) (Left) AKT was immunoprecipitated from T24 cells treated with PI-103 (0.4 μM) or (Right) the isogenic cell lines HT1080-SG1 (NRAS^{wt};NRAS$^{Q61K+ve}$) and SG2 (NRAS^{wt}; loss of NRAS$^{Q61K+ve}$) [10]. Immunoprecipitates and lysates were blotted with the indicated antibodies. (C) Detection of phosphorylated NPM using a pan phospho-AKT substrate antibody in T24 whole cell lysates. Cells were treated as indicated and lysates western blotted with the indicated antibodies. (D) Detection of pS48-NPM by an antibody raised against a synthetic peptide spanning residues 45-56 of NPM (RTVSLGAGAKDE) and incorporating phospho Serine at position 48. The pS48-NPM signal was lost on treatment of the lysate with **λ** Phosphatase, without affecting the levels of total NPM (E) MDA-MB-468 cells cultured in 0.1 (v/v) % FCS were stimulated with EGF (100 ng/ml) and harvested at the indicated time points. Whole cell lysates were probed with the indicated antibodies. (F) Ribbon model of the NPM monomer surface indicating the position of Ser48. Ser48 lies within the conserved AKDE loop which significantly contributes to the interactions at the NPM pentamer:pentamer interface [11]. (G) *Npm*$^{-/-}$*; p53*$^{-/-}$ MEF infected with retrovirus expressing control pBABE vector, pBABE-NPM, pBABE NPM-S48A or pBABE NPM-S48E. Lysates were separated under semi native gel electrophoresis (Top) or denaturing conditions (Bottom) and probed with the indicated antibodies (Dotted line represents spliced image of the same gel) (H) Lysates from cells as in (G) were processed under different denaturing conditions and separated by gel electrophoresis and probed for Nucleophosmin. Panel on the left shows lysates prepared with 1% Nonident (NP) 40 under Native conditions and separated by Native gel electrophoresis. The panel in the middle shows lysates prepared with 2% SDS and separated under semi-native conditions using Bis-Tris gel. Panel on the right shows lysates prepared under denaturing conditions and separated using Bis-Tris gel.

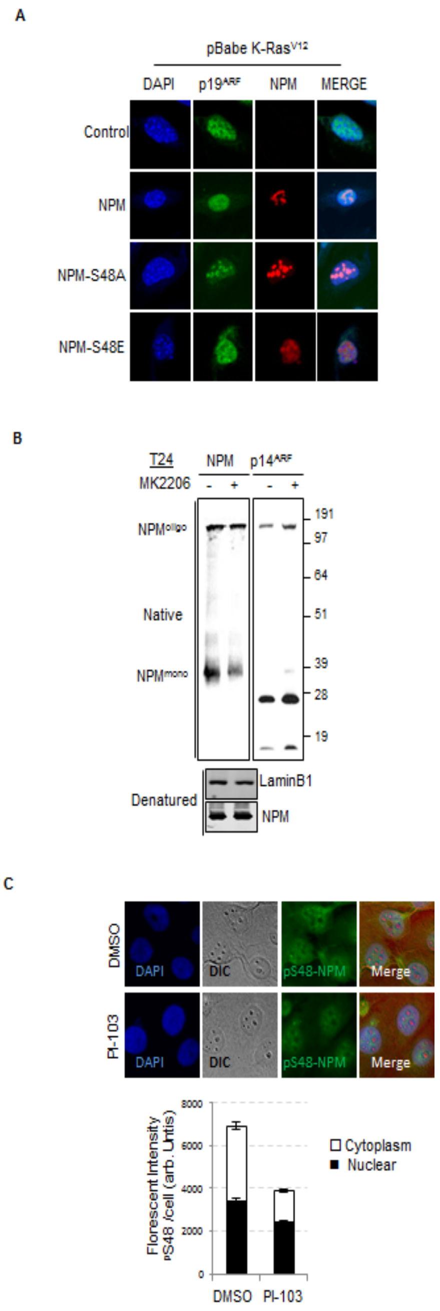

Fig. S2: ARF-NPM co-localization is AKT dependent as is pNPM localization in the cellular compartments. (A) *Npm-/-*, *p53-/-*-double null MEF were infected with pBABE retrovirus expressing control empty vector, NPM-WT, NPM-S48A and S48E as indicated along with K-Ras^{V12} mutant variant. The cells were fixed and stained for p19^{ARF} (green) and NPM (red). (B) Nucleoplasmic fractions from T24 cells that were treated with MK-2206 (5 μM, 24 hrs) were subjected to semi-native gel

electrophoresis and probed by western blot with the indicated antibodies. (C) T24 cells were treated with PI-103 (0.4μM) and stained for pS48-NPM. Contrast microscopy image shows nucleoli and IF image shows pS48-NPM staining (green). Panel below shows bar graph of fluorescence intensity of pS48-NPM staining in the different compartments of the cell as measured by InCell analyzer 1000 automated florescent microscope.

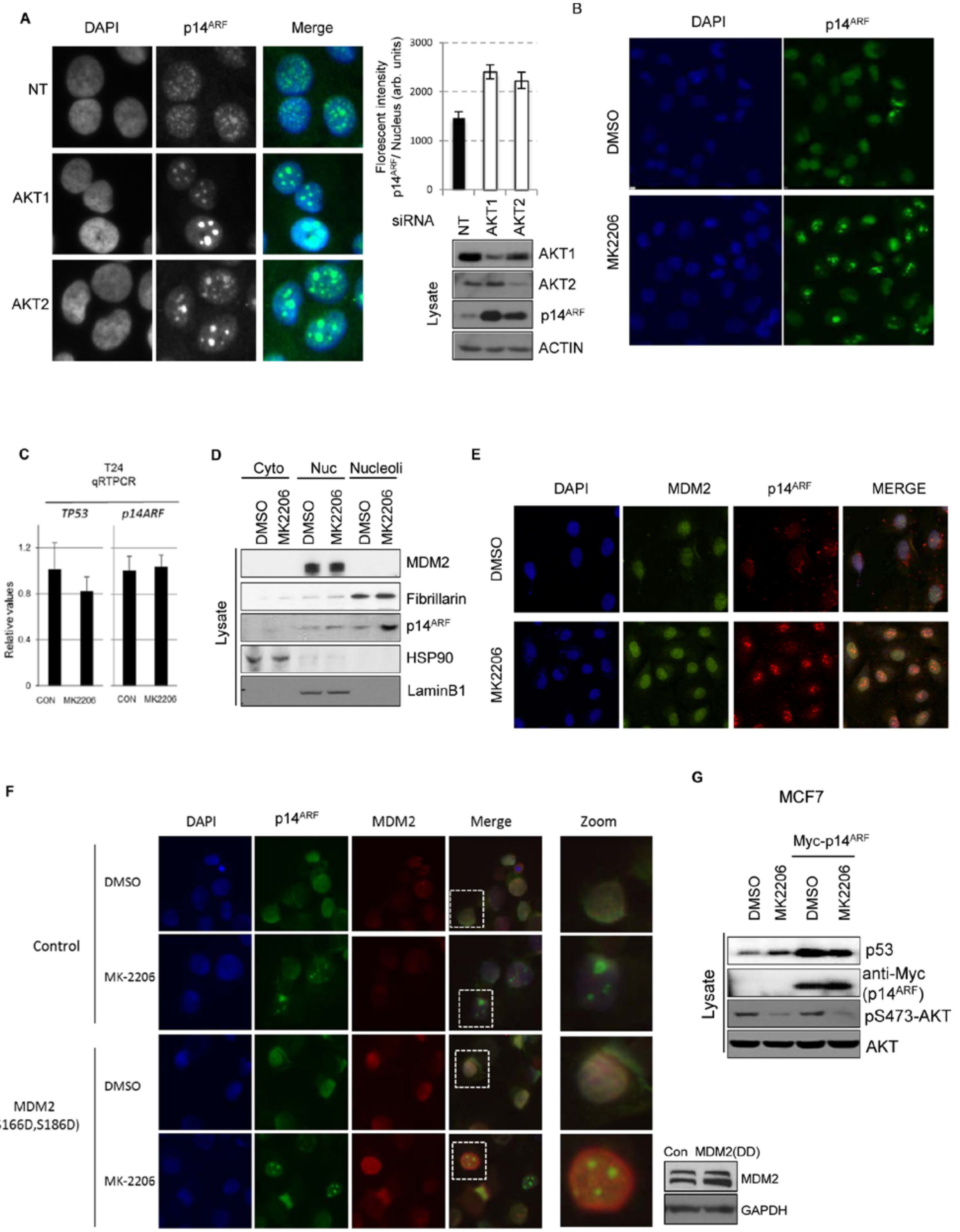

A
DAPI
p14ARF
Merge
NT
AKT1
AKT2
Florescent intensity
p14ARF/ Nucleus (arb. units)
3000
2000
1000
0
siRNA
NT
AKT1
AKT2
Lysate
AKT1
AKT2
p14ARF
ACTIN
B
DAPI
p14ARF
DMSO
MK2206
C
T24
qRTPCR
TP53
p14ARF
Relative values
1.2
0.8
0.4
0
CON
MK2206
CON
MK2206
D
Cyto
Nuc
Nucleoli
DMSO
MK2206
DMSO
MK2206
DMSO
MK2206
Lysate
MDM2
Fibrillarin
p14ARF
HSP90
LaminB1
E
DAPI
MDM2
p14ARF
MERGE
DMSO
MK2206
F
DAPI
p14ARF
MDM2
Merge
Zoom
Control
DMSO
MK-2206
MDM2
(S166D,S186D)
DMSO
MK-2206
Con
MDM2(DD)
MDM2
GAPDH
G
MCF7
Myc-p14ARF
DMSO
MK2206
DMSO
MK2206
Lysate
p53
anti-Myc
(p14ARF)
pS473-AKT
AKT

Fig. S3: AKT regulates ARF localization and ARF-MDM2 interaction in cancer cells. (A)(Left) T24 cells were transfected with siRNA targeted against AKT isoforms 1 or 2 or a non-targeting (NT) control. 48 hrs after transfection, cells were fixed and stained with DAPI and $p14^{ARF}$. (Right upper) Quantitation of $p14^{ARF}$ staining intensity in the nucleus was done using the In Cell Analyzer 1000 automated epifluorescence microscope. Data are represented as mean ± SEM. (Right lower) Whole cell lysates were probed by western blot as indicated. (B) H1299 cells treated with MK-2206 (5µM, 24 hrs) or DMSO and stained for $p14^{ARF}$ (green). (C) Relative levels of p53 or $p14^{ARF}$ mRNA in the presence of MK-2206 (5 µM, 24hrs) determined by qRT-PCR (D) Representative western blots of cytoplasmic, nuclear and nucleolar fractions (obtained by ultracentrifugation of sonicated nucleoplasmic lysates) from T24 cells. Subcellular fractions were probed by western blot with the indicated antibodies. (E) T24 cells were treated with DMSO or MK-2206 (5 µM, 24 hrs) and the localization of $p14^{ARF}$ (red) and MDM2 (green) determined by immunofluorescence. Nuclei were stained with DAPI. (F) T24 cells were transfected with pCDNA3 empty vector or double phopsho-mimetic MDM2 S166D,S186D and treated with DMSO or MK-2206 (5µM). The cells were trypsinised and spun onto glass slides using a Thermo Scientific Cytospin 4 (1200RPM for 4 minutes), fixed and stained for $p14^{ARF}$ (green) and MDM2 (red). Panel on the right shows lysates from the transfected T24 cells probed with the indicated antibodies. (G) MCF7 cells were transfected with Myc tagged $p14^{ARF}$, treated with MK-2206 (5µM,24 hrs) or DMSO and probed by western blot as indicated.

A

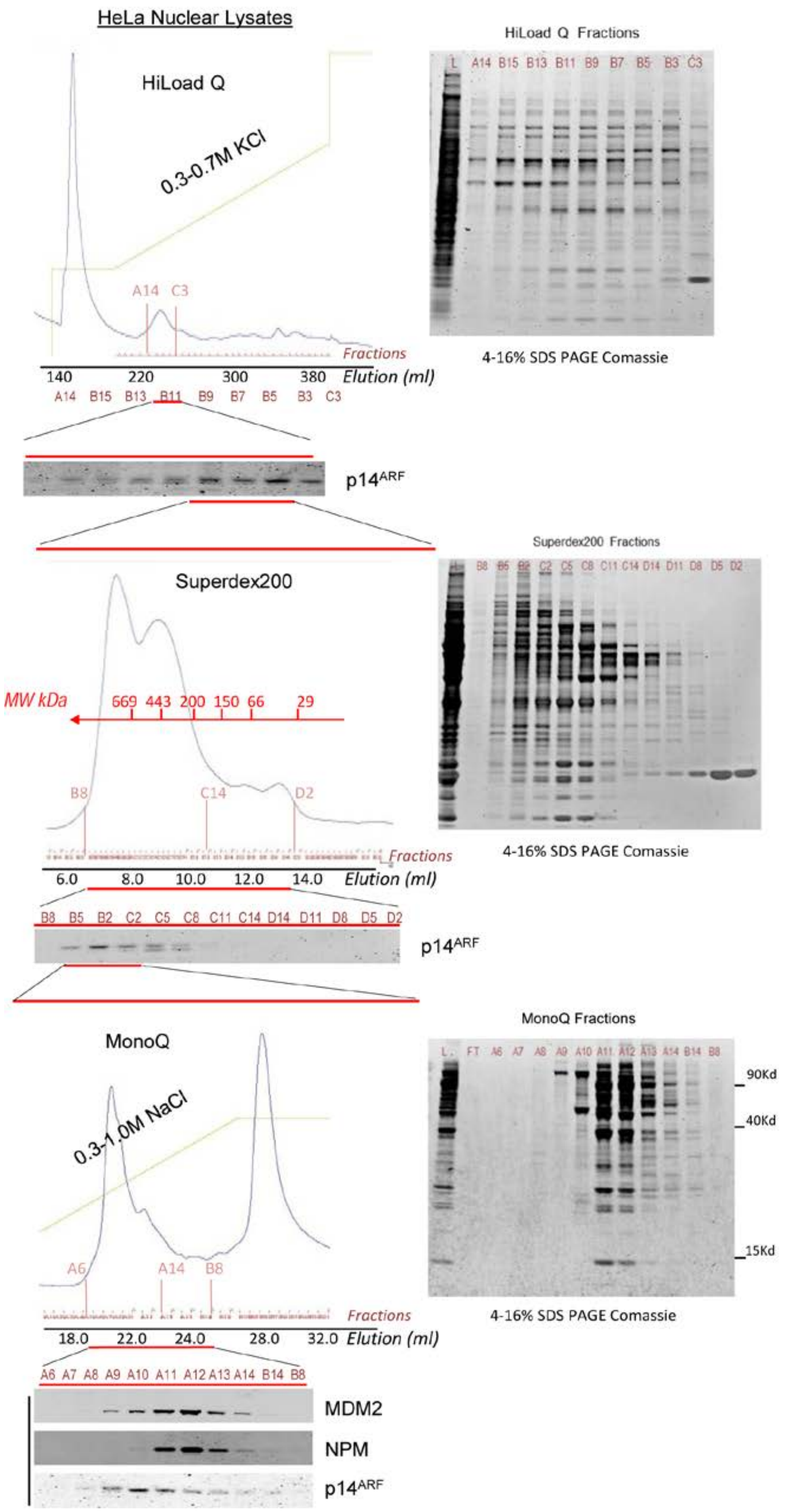
HeLa Nuclear Lysates
HiLoad Q
0.3-0.7M KCl
A14 C3
Fractions
140 220 300 380 Elution (ml)
A14 B15 B13 B11 B9 B7 B5 B3 C3
p14ARF
HiLoad Q Fractions
L A14 B15 B13 B11 B9 B7 B5 B3 C3
4-16% SDS PAGE Comassie
Superdex200
MW kDa
669 443 200 150 66 29
B8 C14 D2
Fractions
6.0 8.0 10.0 12.0 14.0 Elution (ml)
B8 B5 B2 C2 C5 C8 C11 C14 D14 D11 D8 D5 D2
p14ARF
Superdex200 Fractions
4-16% SDS PAGE Comassie
MonoQ
0.3-1.0M NaCl
A6 A14 B8
Fractions
18.0 22.0 24.0 28.0 32.0 Elution (ml)
A6 A7 A8 A9 A10 A11 A12 A13 A14 B14 B8
MDM2
NPM
p14ARF
MonoQ Fractions
L FT A6 A7 A8 A9 A10 A11 A12 A13 A14 B14 B8
90Kd
40Kd
15Kd
4-16% SDS PAGE Comassie

B

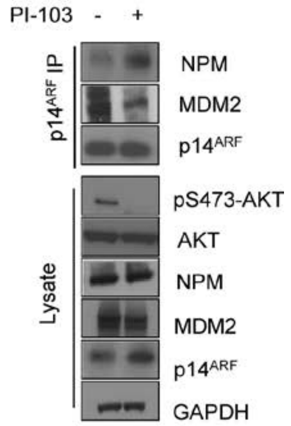
PI-103 - +
p14ARF IP
NPM
MDM2
p14ARF
Lysate
pS473-AKT
AKT
NPM
MDM2
p14ARF
GAPDH

Fig. S4: Identification of NPM interacting proteins on gel separation .(A) p14^{ARF} containing complexes were purified from nuclear lysates prepared from a commercial prep of Hela cells. Lysates were first resolved on a HiLoad Q anion exchange column and proteins eluted with a linear gradient KCl (0.3-0.7 M). p14^{ARF} containing fractions were pooled and further resolved on a Superdex200 gel filtration column. Those fractions containing p14^{ARF} following gel filtration, were pooled and subsequently resolved on a MonoQ anion exchange column. Proteins were eluted with a liner gradient of NaCl (0.3-1.0 M) and fractions containing MDM2, NPM and p14^{ARF} (eluting at approx. 0.5 M NaCl) identified by western blot. Panels on the right show SDS PAGE gels stained with Comassie blue to identify the protein bands. (B) p14^{ARF} was immunoprecipitated from T24 cells treated with PI-103 (0.4 μM) or DMSO. Immunoprecipitates and whole cell lysates were probed with the indicated antibodies.

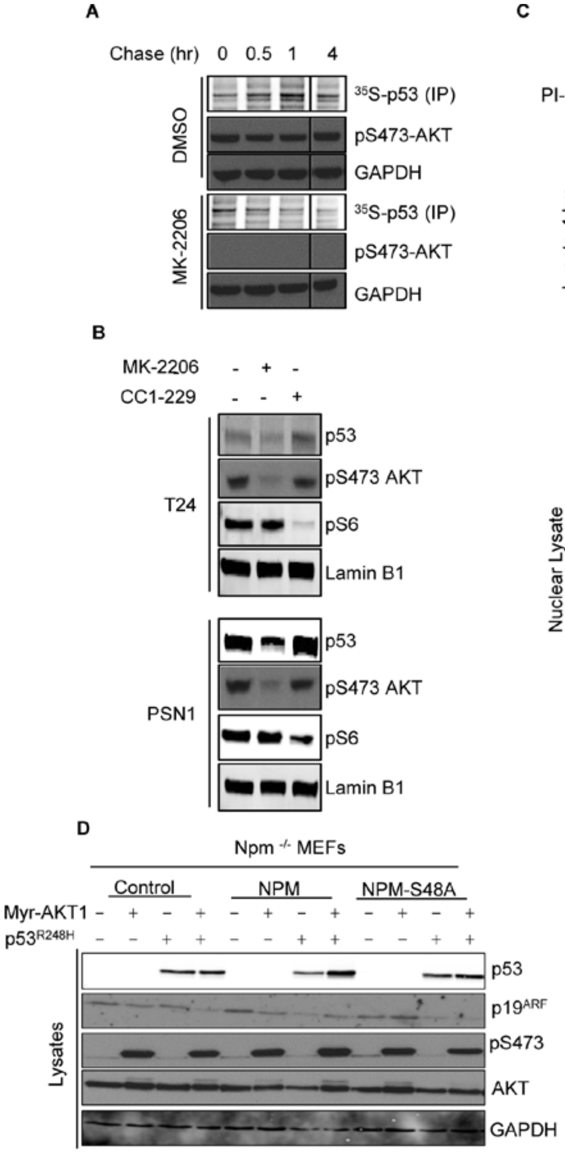

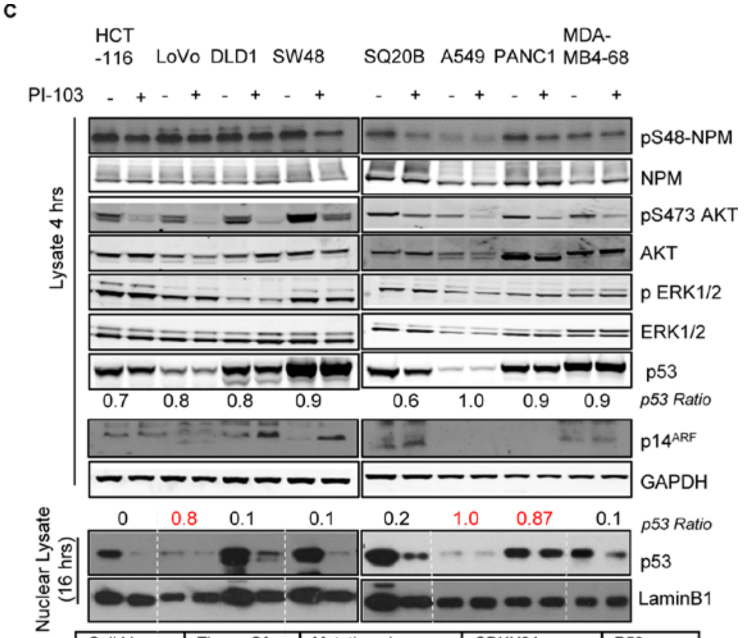

Cell Line	Tissue Of Origin	Mutations in EGFR/RAS/ PI3K/AKT	CDKN2A ARF Status	P53 Status
T24	Bladder	HRAS G12V		Δ Y126
PSN1	Pancreatic	KRAS G12		K162N
HCT-116	Colon	KRAS G12D<br>PIK3CA H1047R		WT
LOVO	Colon	KRAS G13D	Methylated	WT
DLD1	Colon	KRAS G13D		S241F
SW48	Colon	EGFR G719S		R248W
SQ20B	Head and neck	Hyperactive EGFR		insertion Y126
A549	Lung	KRAS G12S	Deleted	WT
PANC1	Pancreas	KRAS G12D	Deleted	R273H
MDA-MB-468	Breast	PTEN		R280K
SKMEL28	Skin	EGFR P753S		L145R

Mutational data from COSMIC (Catalogue Of Somatic Mutations In Cancer) Cell Line Project
http://cancer.sanger.ac.uk/cancergenome/projects/cell_lines/

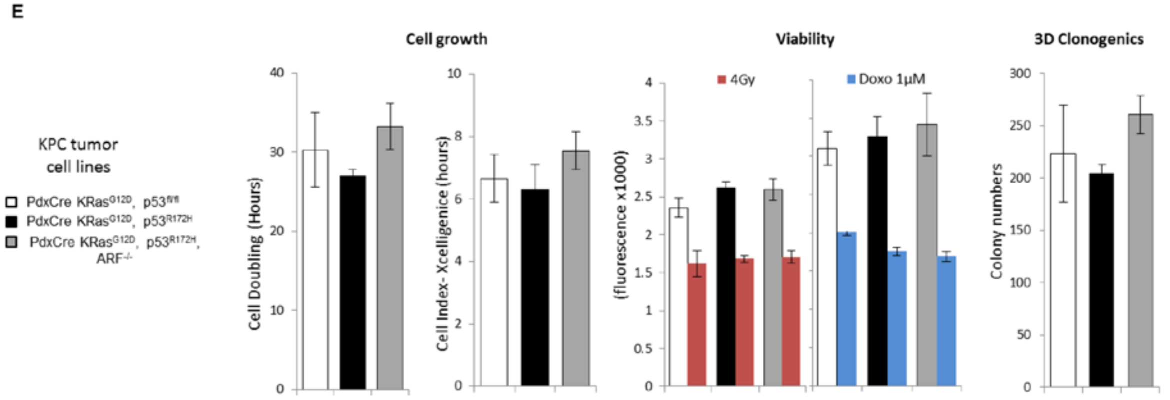

Fig. S5: p53 protein stability is ARF dependent; KPC mouse cell-line survival assays . (A) T24 cells were pre-treated with DMSO or MK-2206 (5 µM,) for 24 hrs before the addition of Met/Cys free media. ^{35}S labeled Met/Cys was then added to the media before being chased for the indicated times with unlabeled Met/Cys. ^{35}S labeled p53 identified by autoradiography of p53 immunoprecipitates. Whole cell lysates were probed with the indicated antibodies (B) T24 cells and PSN1 cells were treated with DMSO control, MK-2206 (5 µM) or CCI-779 (1 µM) for 16 Hrs. Nuclear extracts were prepared from the cells and probed with the indicated antibodies. (C) Cell lines harboring activating mutations in the EGFR-RAS-PI3K -AKT pathway were treated with PI-103 for 4 Hrs (upper) or 16 Hrs (lower). Whole cell (upper) and nuclear lysates were western blotted with the indicated antibodies. Levels of p53 were normalized relative to the GAPDH or Lamin B1 loading control and expressed as a ratio of the p53 levels present in DMSO treated cells. All mutational data were obtained from the COSMIC database http://www.sanger.ac.uk/genteics/CGP/cosmic and ICR (Institute for Cancer Research) database https://cansar.icr.ac.uk/cansar/cell-lines. (D) *Npm-/-*, *p53-/-* double null MEF were infected with pBABE retrovirus expressing control empty vector, NPM-WT and NPM-S48A along with Myr-AKT1 and p53^{R248H} and probed by western blot as indicated. (E) KPC mice derived KRAS^{G12D} p53 Floxed (p53^{Fl}), KRAS^{G12D} p53^{R172H} ARF$^{+/+}$ and KRAS^{G12D} p53^{R172H} ARF$^{-/-}$ pancreatic tumour cell lines were compared for growth, viability and colony forming characteristics. The bar graph on the left of the cell growth panel shows doubling time measured by visual counting of the cells on a haemocytometer at 24 hours after initial seeding of cells and extrapolating the data to give the doubling time in hours. The bar graph on the right shows doubling time of cell index (CI) measured on an xCELLigence RTCA DP analyser (Cell index doubling time = A* 2^(t/CI doubling-time). The panel in the middle shows viability of cells, as measured by fluorescence produced by conversion of Resazurin to Resofurin by viable cells after 48 hours following treatment with 4Gy radiation (bar on left of middle panel) or Doxorubicin (1µM) (bar on right of middle panel). The panel on the right shows 3D colony forming potential of the cells.

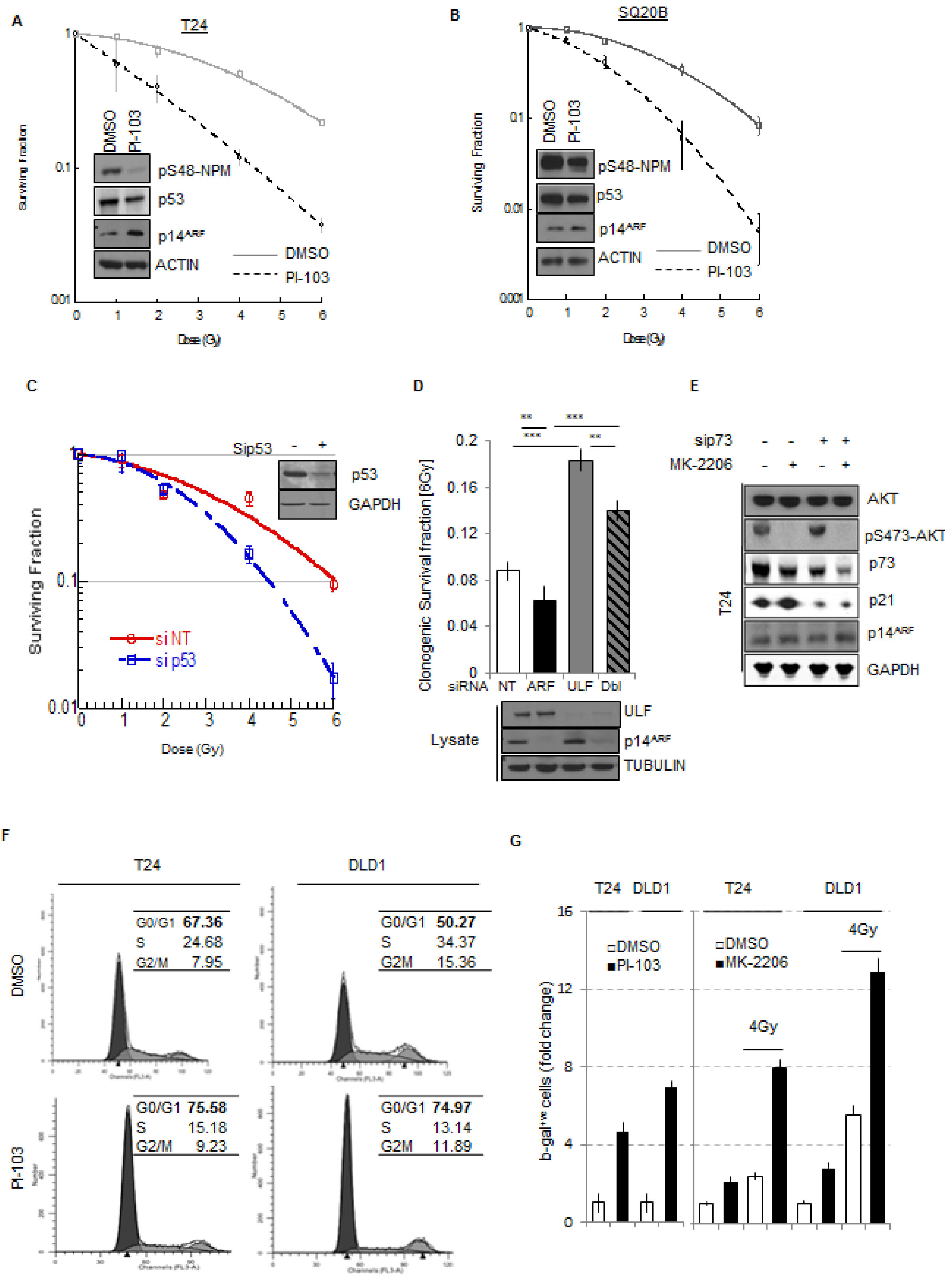

A
T24
Surviving Fraction
Dose (Gy)
DMSO
PI-103
pS48-NPM
p53
p14ARF
ACTIN
B
SQ20B
C
Sip53
GAPDH
si NT
si p53
D
Clonogenic Survival fraction [6Gy]
siRNA NT ARF ULF Dbl
Lysate
ULF
TUBULIN
E
sip73
MK-2206
AKT
pS473-AKT
p73
p21
T24
F
DLD1
G0/G1 67.36
S 24.68
G2/M 7.95
G0/G1 50.27
S 34.37
G2M 15.36
G0/G1 75.58
S 15.18
G2/M 9.23
G0/G1 74.97
S 13.14
G2M 11.89
G
b-gal+ve cells (fold change)
4Gy

Fig. S6: Sensitivity to radiation in mut.p53 cell lines is AKT and p73 dependent and AKT inhibition results in cell cycle arrest and senescence .Clonogenic survival assay and western blots of whole cell lysates of (A)T24 cells and (B) SQ20B cells in the presence of PI-103 (0.4 μM) or DMSO control. (C) T24 cells were transfected with siRNA against p53 or a non-targeting control. (A), (B) and (C) were irradiated at the indicated doses. (D) T24 cells were transfected with siRNA against $p14^{ARF}$ or ULF individually or in combination as indicated. Cells were irradiated (6 Gy) and surviving fraction determined (bars, ** > 0.01; *** > 0.001, student *t*-test). (E) T24 cells transfected with siRNA against p73 or a non-targeting control and were treated with MK-2206 (0.5 μM) for 16 hrs. Whole cell lysates were probed with the indicated antibodies. (F) T24 and DLD1 cells were treated with PI-103 (0.4 μM) or DMSO and stained with propidium iodide prior to FACS analysis of cell cycle profiles. (G) T24 and DLD1 cells were treated with PI-103 (0.4 μM) as above (left) or MK-2206 (5 μM) for 24 hrs and then exposed to single dose of ionizing radiation (IR) (4 Gy). Cells were maintained in culture media in the absence of drug for 5 days and senescent cells determined by staining for β-galactosidase activity. Bars indicate fold increase over background.

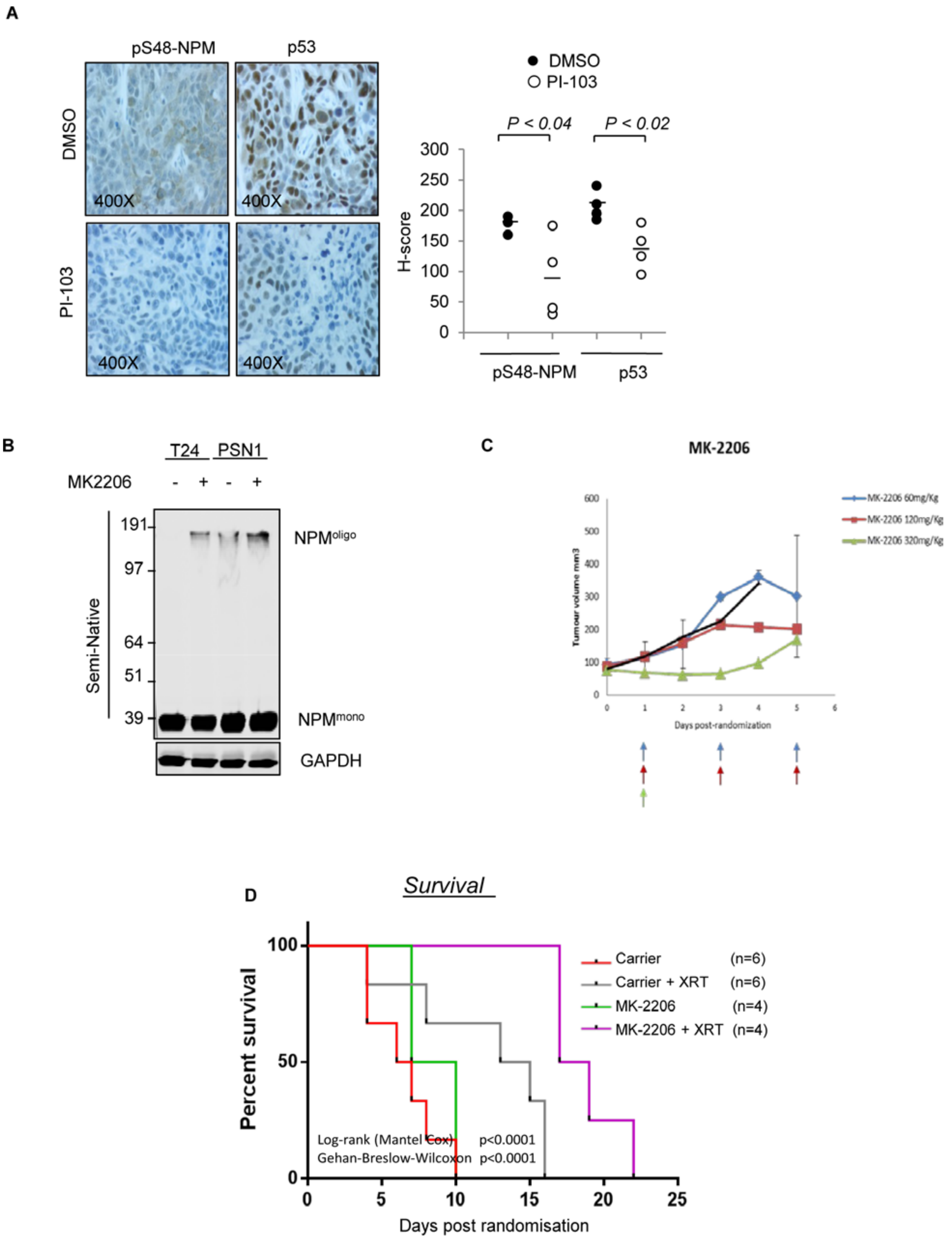
A
pS48-NPM
p53
DMSO
PI-103
400X
DMSO
PI-103
P < 0.04
P < 0.02
H-score
300
250
200
150
100
50
0
pS48-NPM
p53
B
T24
PSN1
MK2206
- + - +
191
97
64
51
39
Semi-Native
NPMoligo
NPMmono
GAPDH
C
MK-2206
MK-2206 60mg/Kg
MK-2206 120mg/Kg
MK-2206 320mg/Kg
Tumour volume mm3
Days post-randomization
D
Survival
Percent survival
100
50
0
Carrier (n=6)
Carrier + XRT (n=6)
MK-2206 (n=4)
MK-2206 + XRT (n=4)
Log-rank (Mantel Cox) p<0.0001
Gehan-Breslow-Wilcoxon p<0.0001
0
5
10
15
20
25
Days post randomisation

Fig. S7: pNPM expression in xenografts and survival data of mice treated with combination therapy . (A) Immunohistochemical staining of p53 and pS48-NPM in adjacent sections of SQ20B xenografts from two independent SCID mice treated with PI-103 or DMSO (400x magnification). Panel on the right shows H-score of staining intensity of sections from individual mice. N=4 for each condition. (B) Semi-native PAGE of whole cell lysates from T24 cells or PSN1 cells treated with MK-2206 (5 μM, 24 hrs). (C) PSN1 xenograft growth curve in mice treated with MK-2206 (60 mg/kg, 120mg/kg and 320 mg/kg). (D) Survival data (4X initial tumour volume) of the mice with PSN1 xenografts treated with two alternative day doses of MK-2206 (60 mg/kg) or β- cyclo-dextrin (1.5 mg/ml) carrier with or without a single dose of IR (6 Gy). Mice were sacrificed when any single dimension reached 12.5 mm.

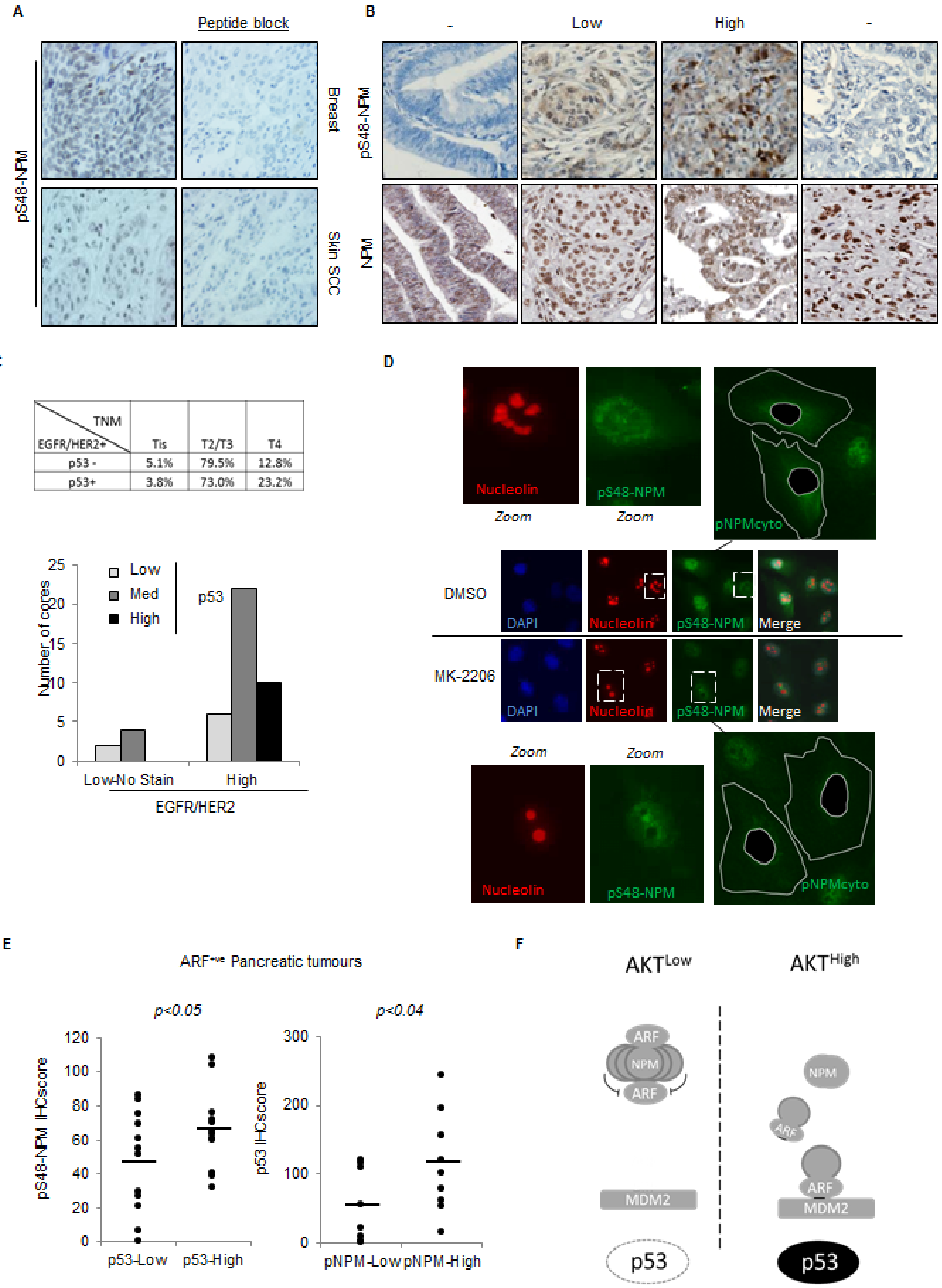

TNM / EGFR/HER2+	Tis	T2/T3	T4
p53 -	5.1%	79.5%	12.8%
p53+	3.8%	73.0%	23.2%

Fig. S8: pS48-NPM antibody validation in xenografts and statistical analysis of p53-pNPM and ARF correlation in human tumours . (A) TMA's (US Biomaxx) were pre-incubated in the presence or absence of the immunogenic peptide used to raise the anti-phospho-Ser48-NPM (pS48-NPM) antibody before staining with pS48-NPM. (B)TMA from pancreatic tumors displayed in Figure 8C, were stained with pS48-NPM (top) or total NPM (bottom). (C) Data from the breast tumor microarray, Figures 8A & 8B, demonstrating the correlation of EGFR/HER2 positivity and low, medium or high p53 staining. (D) T24 cells treated with MK-2206 (5μM, 24 hrs) or DMSO as indicated and stained for Nucleolin and pS48-NPM. Panels show enlarged images of Nucleolin and pS48-NPM staining in cells as well as cytoplasmic pS48-NPM staining in cells where the nuclei in the images have been electronically blacked out to highlight the cytoplasmic staining (E) Tumors from 40 patients with pancreatic tumors were subjected mRNA analysis for $p14^{ARF}$ expression. 26 out of 40 were positive for $p14^{ARF}$ mRNA. Graph indicates correlation of 25 tumors with $p14^{ARF}$ positive signals, splitting population on median and significance verified by student *t*-test (one tailed). (F) Proposed model of AKT mediated phosphorylation of NPM at Serine 48, which regulates its interaction with ARF and MDM2.

Tissue	Lung	Pancreas	Cervix	Colon	Breast
Total (n)	43	40	200	69	144
pS48-NPM$^+$	21	20	130	44	117
% positive	48.8	50	64	63.8	80.7
Low	16	10	70	33	40
Moderate	5	6	31	7	51
High	0	4	29	4	26

Supplementary Table1

		AKT activity			
		pS473	pT308	pS473	pT308
p53 vs AKT activity	Split *CDKN2A* (mRNA)	(<50)		(>50)	
	p53 (protein) correlation	**-0.281**	**-0.284**	-0.092	-0.016
	sig. (2 tailed)	**0.014**	**0.013**	ns	ns
	N	76	76	326	326
	Split *CDKN2A* (mRNA)	(<100)		(>100)	
	p53 (protein) correlation	**-0.276**	**-0.203**	-0.014	0.22
	sig. (2 tailed)	**0.0002**	**0.006**	ns	ns
	N	180	180	222	222
	Split *TP53* (mutation)	(no Mut)		(Mut)	
	p53 (protein) correlation	**-0.236**	**-0.132**	-0.07	-0.069
	sig. (2 tailed)	**4.4 $\times 10^{-5}$**	**0.023**	ns	ns
	N	295	295	107	107

		pS473-AKT	pT308-AKT	p53 (protein)
AKT activity vs AKT substrate	pRAS40 (phospho) correlation	**0.491**	**0.474**	**0.275**
	sig. (2 tailed)	**1 $\times 10^{-13}$**	**1 $\times 10^{-13}$**	**1.99 $\times 10^{-8}$**
	N	402	402	402

		p53 Status					
		all p53		non-Mut		Mutant	
AKT activity vs p53	Split *CDKN2A* (mRNA)	(<50)	(>50)	(<50)	(>50)	(<50)	(>50)
	pRAS40 (protein) correlation	0.017	**0.327**	0.079	**0.240**	-0.248	**0.314**
	sig. (2 tailed)	ns	**1.5 $\times 10^{-9}$**	ns	**0.0002**	ns	**0.002**
	N	76	326	62	233	14	93
	Split *CDKN2A* (mRNA)	(<100)	(>100)	(<100)	(>100)	(<100)	(>100)
	pRAS40 (protein) correlation	0.143	**0.37**	0.134	**0.296**	0.001	**0.314**
	sig. (2 tailed)	ns	**1 $\times 10^{-8}$**	ns	**0.0003**	ns	**0.005**
	N	180	222	152	143	28	79

Supplementary Table 2. AKT activity positively correlates with p53 levels in CDKN2A^{+ve} breast cancer.

The correlation of p53 protein levels with the activity of AKT was studied in 402 breast cancer patients from the TCGA database for whom complete information on protein and gene expression levels together with *TP53* mutational status was available. The data was downloaded from cBioPortal for Cancer Genomics [1,2] and analysed with SPSS 21.0 software. The Shapiro-Wilk test was used to assess distribution of datasets, whereby the null hypothesis of normal distribution was rejected for all datasets tested ($p<0.05$). Correlation analysis was carried out using the non-parametric Spearman-Rho test.

1. Cerami, E., Gao, J., Dogrusoz, U., Gross, B. E., Sumer, S. O., Aksoy, B. A., Jacobsen, A., Byrne, C. J., Heuer, M. L., Larsson, E., *et al.* (2012). The cBio cancer genomics portal: an open platform for exploring multidimensional cancer genomics data. Cancer discovery *2*, 401-404.
2. Gao, J., Aksoy, B. A., Dogrusoz, U., Dresdner, G., Gross, B., Sumer, S. O., Sun, Y., Jacobsen, A., Sinha, R., Larsson, E., *et al.* (2013). Integrative analysis of complex cancer genomics and clinical profiles using the cBioPortal. Science signaling *6*, pl1.
